# Temperature-controlled repeatable scrambling and induced-sorting of building blocks between cubic assemblies

**DOI:** 10.1038/s41467-019-09495-1

**Published:** 2019-03-29

**Authors:** Yi-Yang Zhan, Tatsuo Kojima, Kentaro Ishii, Satoshi Takahashi, Yohei Haketa, Hiromitsu Maeda, Susumu Uchiyama, Shuichi Hiraoka

**Affiliations:** 1 Department of Basic Science, Graduate School of Arts and Sciences, The University of Tokyo, 3-8-1 Komaba, Meguro-ku, Tokyo, 153-8902 Japan; 2 Exploratory Research Center on Life and Living Systems (ExCELLS), National Institutes of Natural Sciences, 5-1 Higashiyama, Myodaiji-cho, Okazaki, Aichi, 444-8787 Japan; 3 Department of Applied Chemistry, College of Life Sciences, Ritsumeikan University, 1-1-1 Noji-higashi, Kusatsu, Shiga, 525-8577 Japan; 4 Department of Biotechnology, Graduate School of Engineering, Osaka University, 2-1 Yamadaoka, Suita, Osaka, 565-0871 Japan

## Abstract

Separation of a homogeneous mixture of different components to reach an ordered out-of-equilibrium state in solution has attracted continuous attention. While this can be achieved using external chemical fuels or photo energy, an alternative energy source is heat. Here we realize a temperature-controlled cycle of transitions between ordered and disordered states based on a mixture of two kinds of building blocks that self-assemble into cubic structures (nanocubes). An almost statistical mixture of nanocubes (disordered state) is thermodynamically most stable at lower temperature (25 °C), while homoleptic assemblies composed of single components are preferentially produced at higher temperature (100 °C) followed by rapid cooling. The scrambling of the building blocks between the nanocubes takes place through the exchange of free building blocks dissociated from the nanocubes. Based on this mechanism, it is possible to accelerate, retard, and perfectly block the scrambling by the guest molecules encapsulated in the nanocubes.

## Abstract (web summary)

In this paper, the authors study the temperature-controlled dynamic behavior of a system of nanocubes self-assembled from two different building blocks. Non-intuitively, the disordered, equilibrium state (a mixture of heteroleptic cubes) and the ordered, out-of-equilibrium state (a mixture of homoleptic cubes) are cycled by heating and subsequent rapid cooling.

## Introduction

Two kinds of different gases put into a box spontaneously mix together to reach a homogeneous fluid in equilibrium. Such a disordered state is thermodynamically favored and entropy is the quantity that determines which direction a reaction spontaneously proceeds to. Thus, the separation of each component from a homogeneous mixture (towards ordered state) needs external energy or help of Maxwell’s demon if we want to realize the separation without any energy except for information^1–4^. The homogeneity of a mixture of gases in equilibrium derives from their negligibly weak interactions. Contrary to mixing of gases, molecular self-assembly of multiple components can spontaneously form homoleptic self-assemblies composed of single components through self-sorting under thermodynamic control with the aid of intermolecular interactions between well-designed molecular building blocks that discriminate between oneself and others^5–19^. Furthermore, in the case where a statistical mixture of components in molecular assemblies is thermodynamically preferred, recent progress on adaptive chemistry has realized the transition of such disordered states into an ordered out-of-equilibrium state by external photo or chemical energy^20–34^.

Here we present a simple example of a temperature-controlled reversible transition between ordered and disordered states consisting of a mixture of two kinds of molecular building blocks that self-assemble into hexameric cubic structures, i.e. nanocubes, with different thermal stabilities. Because of a structural similarity between the two components (A and B for example), scrambling of the building blocks between the nanocubes (A_6_ and B_6_) spontaneously takes place at 25 °C to reach an almost statistical mixture of nanocubes composed of two kinds of building blocks, A_*n*_B_6–*n*_ (*n* = 0–6), a disordered state. Then, a metastable ordered state (a mixture of homoleptic nanocubes composed of a single component, A_6_ and B_6_) is spontaneously recovered by heating at 100 °C and subsequent rapid cooling. The reversible transition between the two states is realized by the change in the energy landscape of the system consisting of two kinds of building blocks in response to the temperature. It is also found that the scrambling is accelerated, retarded, or blocked by guest molecules encapsulated in the nanocubes, which enables us to lock and unlock a metastable state by the encapsulation and release of hydrophobic molecules in the nanocubes.

## Results

### Scrambling between nanocubes

**Fig. 1:**
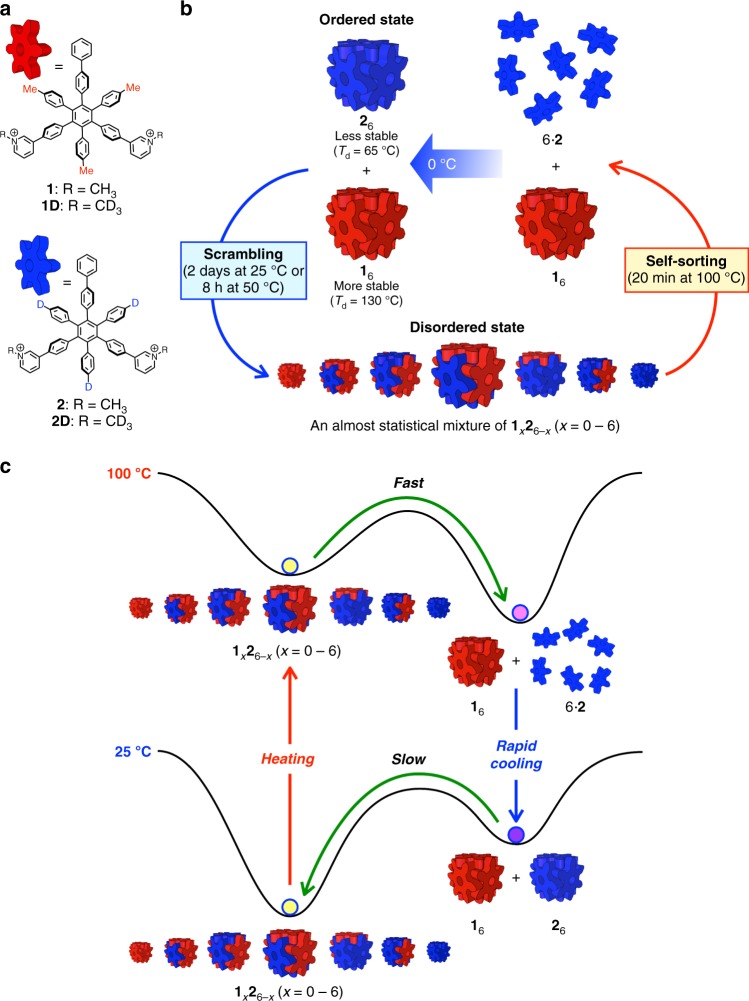
A temperature-controlled cycle of transitions between ordered and disordered states in a mixture of nanocubes (**1**_6_ and **2**_6_). **a** The chemical structures of GSAs used in this research. **b** A temperature-controlled cycle of the conversion between the ordered and disordered states. An almost statistical mixture of the nanocubes (disordered state) was produced at 25 or 50 °C, while a mixture of two kinds of nanocubes composed of single GSAs was preferentially produced by heating at 100 °C followed by rapid cooling at 0 °C. There are positional isomers for **1**_*x*_**2**_6−*x*_ (*x* = 2–4). **c** A schematic representation of change in the energy landscape of a mixture of two kinds of GSAs, **1** and **2**, in response to the temperature. An almost statistical mixture of nanocubes **1**_*x*_**2**_6–*x*_ (*x* = 0–6) is thermodynamically most stable at 25 °C, while a mixture of **1**_6_ and monomer **2** is thermodynamically most stable at 100 °C because decomposition temperatures of the nanocubes composed of more GSAs **2** are lower than 100 °C

**Fig. 2:**
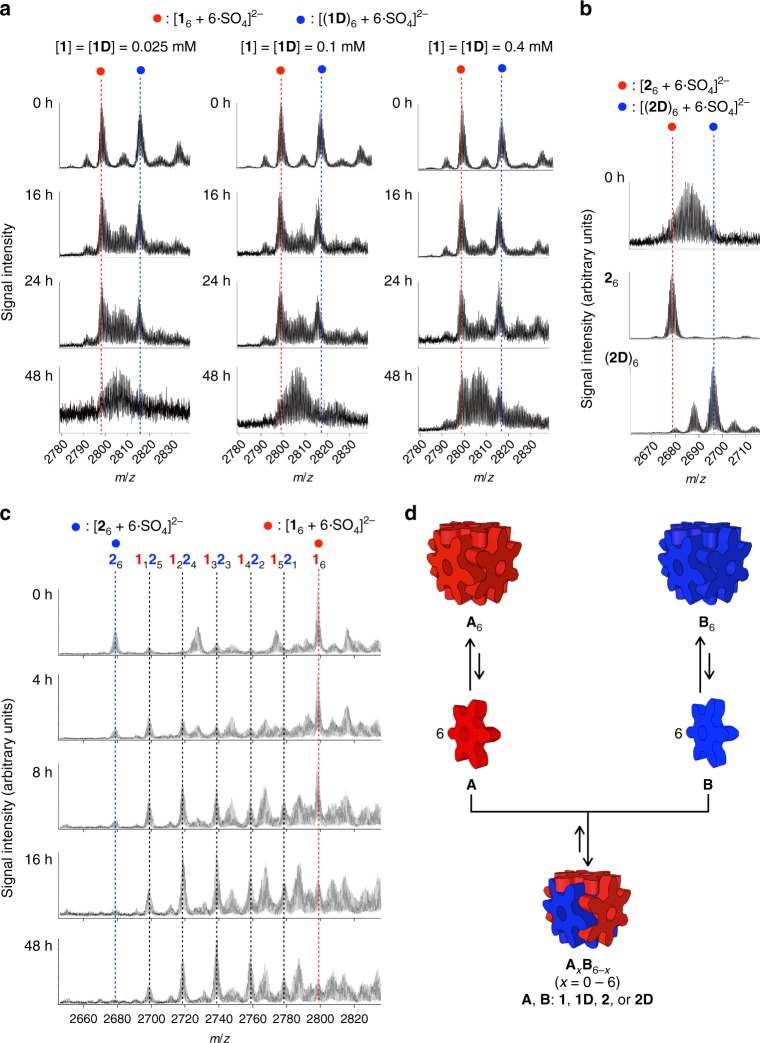
Monitoring of scrambling of GSAs between the nanocubes by ESI-TOF mass spectrometry and scrambling mechanism. **a** The scrambling of GSAs between **1**_6_ and (**1D**)_6_ was monitored in different concentrations. Right after the mixing of aqueous solutions of **1**_6_ and (**1D**)_6_, strong signals for the homoleptic nanocubes (indicated by solid blue and red circles) were observed. These signals became weak with time and new signals assigned to heteroleptic nanocubes appeared in between the two signals. The scrambling takes place faster in lower concentration of the nanocubes. **b** The scrambling of GSAs between **2**_6_ and (**2D**)_6_. The scrambling was completed right after the mixing of **2**_6_ and (**2D**)_6_, which is much faster than the scrambling between **1**_6_ and (**1D**)_6_. **c** The scrambling of GSAs between **1**_6_ and **2**_6_ was monitored after mixing of aqueous solutions of **1**_6_ and **2**_6_. The conversion of the less stable nanocube (**2**_6_) into heteroleptic nanocubes, **1**_*x*_**2**_6−*x*_ (*x* = 1–5), is faster than that of **1**_6_. **d** The scrambling of GSAs between the nanocubes **A**_6_ and **B**_6_ takes place through the exchange of monomer GSAs dissociated from the nanocubes. After the formation of heteroleptic nanocubes **A**_*x*_**B**_6−*x*_ (*x* = 1–5), the scrambling of **A**_*x*_**B**_6−*x*_ (*x* = 1–5) with the less stable homoleptic nanocube takes place faster than with the more stable one

Gear-shaped amphiphiles (GSA: **1**·Cl_2_ and **2**·Cl_2_), which are *C*_2v_-symmetric hexaphenylbenzene (HPB) derivatives possessing three kinds of substituents on the periphery of HPB, self-assemble into a cube-shaped structure (nanocubes, **1**_6_ and **2**_6_) in water driven by the hydrophobic effect and van der Waals (vdW) and cation–π interactions between GSA molecules (Fig. 1a)^35–37^. Though the structures of **1** and **2** are very similar, **1**_6_ is thermally more stable than **2**_6_ because vdW interactions around *p*-tolyl methyl groups of **1**_6_ significantly enhance its thermal stability (disassembly temperature (*T*_d_) of **1**_6_ is 130 °C, while that of **2**_6_ is 65 °C). To investigate the relation between the thermal and kinetic stabilities of the nanocubes, scrambling of GSAs between nanocubes assembled from non-deuterated GSAs (**1**_6_ or **2**_6_) and from partially deuterated GSAs ((**1D**)_6_ or (**2D**)_6_) was monitored by electrospray ionization-time-of flight (ESI-TOF) mass spectrometry. When aqueous solutions of **1**_6_ and (**1D**)_6_ were mixed at room temperature ([**1**] = [**1D**] = 0.025 mM), the scrambling was clearly monitored by change in the distribution of mass signals for [**1**_*x*_(**1D**)_6–*x*_(SO_4_)_6_]^2–^ (*x* = 0–6), which captured two electrons during the ionization (an aqueous H_2_SO_4_ solution was added just before the mass measurement in order to detect the nanocubes as negative ion species), and an almost statistical signal pattern was observed in 2 days (Fig. 2a). In contrast, the scrambling for a mixture of thermally less stable nanocubes, **2**_6_ and (**2D**)_6_, finished just after the mixing of the two solutions (Fig. 2b), indicating that the rate of the scrambling is related to the thermal stability of the nanocubes. It was also found that the rate of the scrambling is dependent on the concentration; the scrambling took place faster at lower concentration of the nanocubes (Fig. 2a). This result indicates that the scrambling does not occur by the collision of the nanocubes but through the exchange of monomer GSAs dissociated from the nanocubes (Fig. 2d) even though the signals for free GSAs were not observed by ^1^H NMR spectroscopy.

**Fig. 3:**
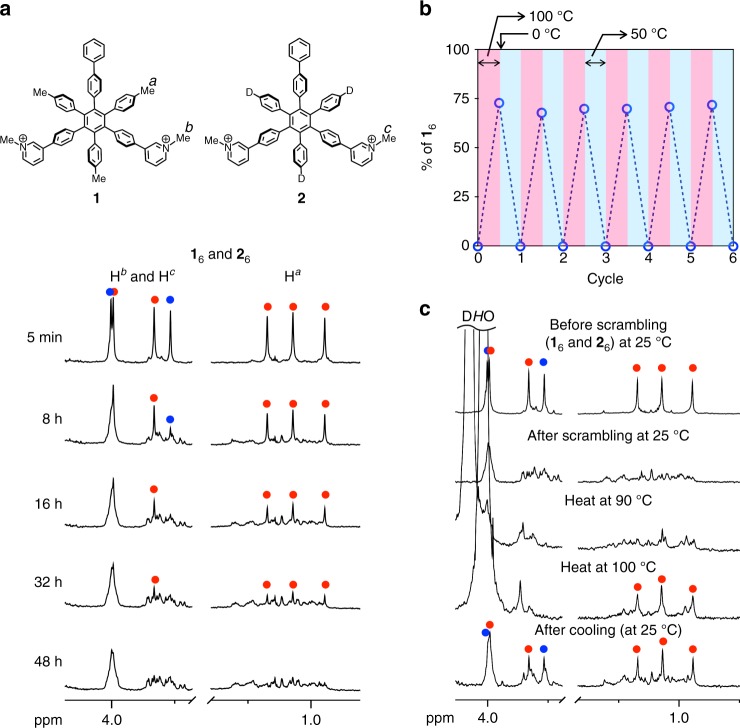
Scrambling and induced-sorting of GSAs between the nanocubes (**1**_6_ and **2**_6_). The scrambling and induced-sorting were monitored by ^1^H NMR spectroscopy (500 MHz, D_2_O, *p*-tolyl methyl and *N*-methyl group regions, [**1**] = [**2**] = 0.4 mM). **a** The monitoring of the scrambling of GSAs between **1**_6_ and **2**_6_ at 298 K. Red and blue solid circles indicate the signals for **1**_6_ and **2**_6_, respectively. **b** A plot of the existence ratio of **1**_6_ in a 1:1 mixture of **1** and **2** during six cycles of the transitions between the ordered and disordered states by changing temperature. **c** ^1^H NMR monitoring of the transition in the first cycle. Red and blue solid circles indicate the signals for **1**_6_ and **2**_6_, respectively. The temperatures at which the measurements were carried out are indicated in the spectra

Next, the scrambling between the two different nanocubes (**1**_6_ and **2**_6_) was monitored by ESI-TOF mass spectrometry (Fig. 2c). When **1**_6_ and **2**_6_ ([**1**] = [**2**] = 0.4 mM) were mixed at 25 °C, the signal for **2**_6_ decreased faster than that for **1**_6_ and finally reached an almost statistical compositional mixture of the nanocubes in 2 days. The scrambling in the same concentration of the nanocubes ([**1**] = [**2**] = 0.4 mM) was monitored by ^1^H NMR spectroscopy (Fig. 3a and Supplementary Fig. 3). The ^1^H NMR spectra of a 1:1 mixture of **1**_6_ and **2**_6_ at 25 °C changed for 2 days, which is consistent with the time taken for the convergence of mass signals of a mixture of **1**_6_ and **2**_6_. As observed by the mass measurements, the ^1^H NMR signals for **2**_6_ decreased faster than those for **1**_6_, indicating that the heteroleptic nanocubes, **1**_*x*_**2**_6−*x*_ (*x* = 1–5), tend to preferentially exchange GSAs with **2**_6_ because the scrambling between less stable nanocubes takes place faster, which supports the idea that the scrambling takes place through the exchange of monomer GSAs dissociated from the nanocubes. The scrambling was accelerated by heating and reached convergence in 8 h at 50 °C.

### Interconversion between ordered and disordered states

Considering the fact that **1**_6_ (*T*_d_ = 130 °C) is thermally more stable than **2**_6_ (*T*_d_ = 65 °C) due to the vdW interactions around *p*-tolyl methyl groups in **1**, nanocubes composed of more **2** would be less stable than those composed of more **1**. Hence, at higher temperature than *T*_d_ of **2**_6_, the nanocubes composed of more **2** should be disassembled to lead to a mixture of monomer **2** and the nanocubes composed of more **1**. Then rapid cooling of the mixture of the nanocubes (mainly **1**_6_) and free **2** thus obtained leads to a mixture mainly containing homoleptic nanocubes (**1**_6_ and **2**_6_) through faster self-assembly of monomer **2** into **2**_6_ than the scrambling (Fig. 1b, c). As expected, heating of a mixture of **1**_6_ and **2**_6_ at 100 °C led to the homoleptic **1**_6_ in 83% yield (Fig. 3c and Supplementary Fig. 5). Subsequent rapid cooling at 0 °C gave homoleptic nanocubes (ordered state) in 70.7 ± 0.7% yield (Fig. 3c and Supplementary Fig. 6). At 50 °C, this mixture was gradually converted into an almost statistical mixture of nanocubes **1**_*x*_**2**_6–*x*_ (*x* = 0–6) (disordered state), which is thermodynamically most stable at 50 °C. The interconversion between the ordered and disordered states is repeatable simply by changing the temperature of the solution without any degradation because the GSAs are completely stable even at 100 °C (Fig. 3b).

### Guest effect on scrambling

**Fig. 4:**
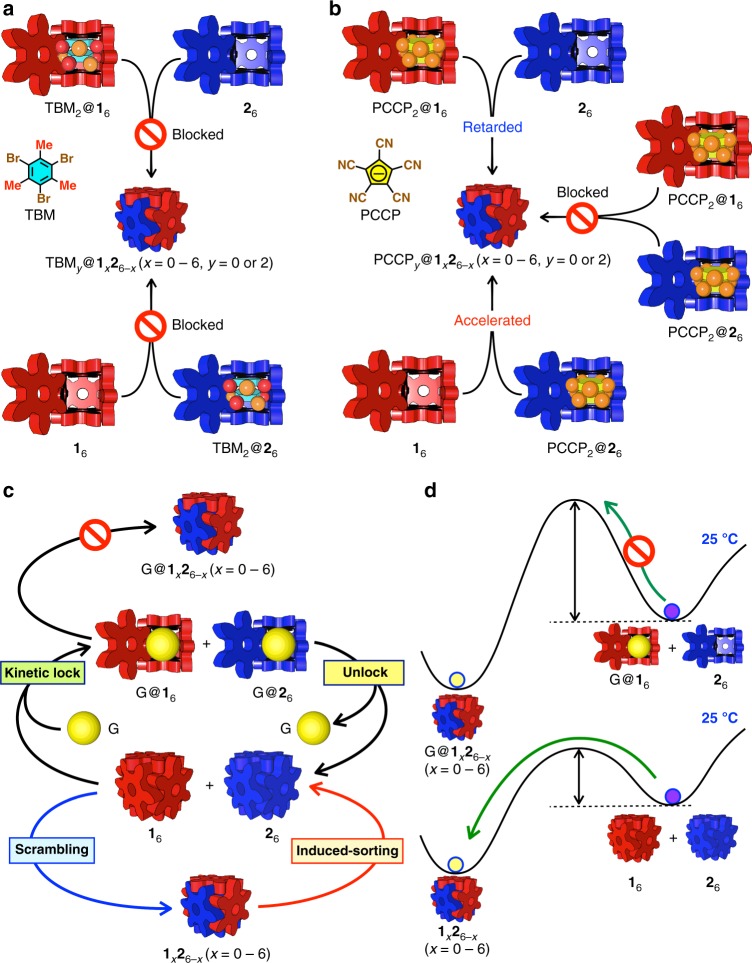
The effect of guest molecules on the scrambling of GSAs between the nanocubes (**1**_6_ and **2**_6_). **a** The encapsulation of hydrophobic guest molecules (TBM: 1,3,5-tribromomesitylene) in **1**_6_ or **2**_6_ blocked the scrambling. **b** Anionic guest molecule (PCCP: pentacyanocyclopentadienide) accelerated, retarded, and blocked the scrambling depending on the way of the encapsulation of PCCP in the nanocubes. **c** A cycle of the transition between the ordered and disordered states coupled with the kinetic lock and unlock of the ordered state by the encapsulation and release of guest molecules (G) in the nanocubes. G indicates two or three molecules of *n*-hexanes. Three molecules of *n*-hexanes are encapsulated in **1**_6_, while two in **2**_6_. The removal of G from the nanocubes was carried out by heating at 100 °C. **d** A schematic representation of change in the energy landscape of the scrambling of GSAs with hydrophobic guest molecules (G). A mixture of G@**1**_6_ and **2**_6_ is kinetically stable at 25 °C (**a**) because the encapsulated hydrophobic guest molecules stabilize the nanocube, giving rise to high activation energy of the scrambling. The same effect works in the cases where the scrambling starts from a mixture of **1**_6_ and G@**2**_6_ (**a**) and from a mixture of G@**1**_6_ and G@**2**_6_ (**c**)

As the nanocubes can encapsulate neutral and anionic species in their about-1-nm-sized hydrophobic inner space to lead to thermally more stable nanocubes^37^, the guest species would prevent the dissociation of the nanocubes into the monomers, thereby retarding the scrambling. When aqueous solutions of **1**_6_ encapsulating two molecules of 1,3,5-tribromomesitylenes (TBM), TBM_2_@**1**_6_ (*T*_d_ > 150 °C), and of **2**_6_ (*T*_d_ = 65 °C) were mixed, nothing happened for 6 months (Supplementary Fig. 7); the scrambling of GSAs and the distribution of TBM molecules between the nanocubes were perfectly blocked (Fig. 4a). The same result was found when aqueous solutions of TBM_2_@**2**_6_ (*T*_d_ = 135 °C) and **1**_6_ (*T*_d_ = 130 °C) were mixed (Supplementary Fig. 8). These results indicate that the encapsulated hydrophobic guest molecules stabilize the nanocubes to change the energy landscape, resulting in the suppression of the dissociation of free GSAs (Fig. 4d). Interestingly, when insoluble TBM existed in an aqueous solution of TBM_2_@**2**_6_ and **1**_6_, the scrambling took place slowly. (It took 26 days to reach equilibration, which is longer than the time for the scrambling between **1**_6_ and **2**_6_, 2 days.) (Supplementary Fig. 9). Insoluble TBM molecules were gradually encapsulated in **1**_6_, during which the structure of the **1**_6_ nanocube would partially be broken or free GSAs would be dissociated. Hence, these (partially) dissociated GSAs of **1** should promote the scrambling. On the other hand, no scrambling took place when insoluble TBM was present in a solution of TBM_2_@**1**_6_ and **2**_6_, though gradual encapsulation of TBM in **2**_6_ was observed (Supplementary Fig. 10). In this case, though the encapsulation of TBM into **2**_6_ caused the dissociation of monomer GSA **2**, the dissociation of monomer GSA **1** was strongly prevented by the encapsulation of TBM into **1**_6_ (*T*_d_ > 150 °C), so the scrambling did not take place. These results indicate that whether the scrambling takes place or not is determined by the existence of monomer GSAs (**1** and **2**) and that in the case where the thermal stabilities of the two nanocubes are significantly different the rate-determining step of the scrambling is the dissociation of monomer GSAs from a thermally more stable nanocube.

Quite different results were observed when anionic species (NaPCCP: sodium pentacyanocyclopentadienide) was used as a guest molecule (Fig. 4b). When PCCP_2_@**1**_6_ and **2**_6_ were mixed together, the scrambling was significantly retarded (65% of PCCP_2_@**1**_6_ and 20% of **2**_6_ existed in the solution after 1 month) (Supplementary Fig. 11). Furthermore, the scrambling was perfectly blocked by the encapsulation of PCCP in both of the nanocubes (Supplementary Fig. 12). Surprisingly, the addition of 2 eq of PCCP in a 1:1 mixture of **1**_6_ and **2**_6_ accelerated the scrambling (the scrambling finished in 1 day, which is faster than that carried out in the absence of PCCP (2 days)) (Supplementary Fig. 13). Right after the addition of PCCP in a solution of **1**_6_ and **2**_6_, all PCCP molecules were selectively encapsulated in thermally less stable **2**_6_ under kinetic control. Then the exchange of PCCP between the nanocubes coincided with the scrambling of GSAs between the nanocubes. A simple mixing of **1**_6_ and PCCP_2_@**2**_6_ also accelerated the scrambling (1 day) (Supplementary Fig. 14). The acceleration of the scrambling under this condition is ascribed to the dissociation of monomer **1** initiated by the transfer of PCCP encapsulated in **2**_6_ into **1**_6_ (PCCP_2_@**2**_6_ + **1**_6_ $\rightarrow$ PCCP_2_@**1**_6_ + **2**_6_).

Because of the lower stability of the ordered state at 25 °C, spontaneous scrambling takes place to reach the disordered state. To control the timing of the scrambling, a guest-encapsulation-release system was coupled with the cycle of transitions (Fig. 4c and Supplementary Fig. 15). After the formation of a mixture of homoleptic nanocubes from the disordered state, a hydrophobic guest molecule (Hex: *n*-hexane) was added in the mixture to form the nanocubes that encapsulate the guest molecules, Hex_3_@**1**_6_ and Hex_2_@**2**_6_. The scrambling of the GSAs between the two nanocubes was blocked for 7 days, indicating that the metastable ordered state was kinetically locked. The removal of the guest molecules by heating at 100 °C is the trigger to unlock the ordered state to lead to a mixture of guest-free homoleptic nanocubes, **1**_6_ and **2**_6_, which then was converted into the disordered state again.

## Discussion

In conclusion, a temperature-controlled cycle of transitions that is fabricated from two kinds of building blocks that self-assemble into homoleptic and heteroleptic cubic assemblies (nanocubes) with different thermal stability was realized. The scrambling experiments (intermolecular exchange) of the building blocks (GSAs) between the nanocubes indicate that the kinetic stability of the nanocube is closely related to the thermal stability of the nanocubes; a thermally more stable nanocube is kinetically more inert. The concentration effect on the rate of the scrambling and the results obtained from the scrambling experiments in the presence of guest molecules indicate that the scrambling takes place through the exchange of free GSAs dissociated from the nanocubes and that the rate of the scrambling is determined by the dissociation of free GSAs provided from a thermally more stable nanocube. The interconversion between the metastable ordered (a mixture of **1**_6_ and **2**_6_) and the thermodynamically most stable disordered states (an almost statistical mixture of **1**_*x*_**2**_6–*x*_ (*x* = 0–6)) was realized simply by changing the temperature. The result that the induced-sorted state was preferred by heating at 100 °C and subsequent rapid cooling was realized because only the nanocubes composed of more **1** can survive at this temperature. The rate of the scrambling can be controlled by anionic guest species. The blocking of the scrambling by the encapsulation of guest molecules would enable to kinetically capture a certain metastable species that is selected from a dynamic combinatorial library of compositional and positional isomers of nanocubes consisting of several kinds of GSAs in response to external stimuli.

## Methods

### General

^1^H and ^13^C NMR spectra were recorded using a Bruker AV-500 (500 MHz) spectrometer. High-resolution mass spectra (HRMS) were obtained using a Waters Xevo G2-S QTOF mass spectrometer. All reagents were obtained from commercial suppliers (TCI Co., Ltd., WAKO Pure Chemical Industries Ltd., KANTO Chemical Co., Inc., and Sigma-Aldrich Co.) and were used as received. GSAs **1**·Cl_2_ and **2**·Cl_2_ and compounds **3** and **4** were synthesized according to previously reported procedures^35^. See supplementary information for synthetic procedures, including Supplementary Figs. 16–19.

### Host–guest complexation

The solutions of TBM_2_@**1**_6_, TBM_2_@**2**_6_ (TBM indicates 1,3,5-tribromomesitylene), PCCP_2_@**1**_6_ (PCCP indicates pentacyanocyclopentadienide) and Hex_3_@**1**_6_ (Hex indicates *n*-hexane) were prepared according to the literatures^35, 37^. Hex (1 μL) was added by a syringe to a D_2_O solution of **2**_6_ ([**2**] = 1.0 mM, 600 μL) in an NMR tube. The suspension was mixed by inverting the NMR tube four times and sonicated for 5 min to afford Hex_2_@**2**_6_ (Supplementary Fig. 1). The formation of PCCP_2_@**2**_6_ was confirmed by the titration experiments using the ^1^H NMR signals of the nanocube encapsulating PCCPs (Supplementary Fig. 2).

### Monitoring of scrambling by ESI-TOF mass spectrometry

ESI-TOF mass spectra of the scrambling of the nanocubes in water were recorded on a SYNAPT G2-S*i* HDMS mass spectrometer (Waters, Massachusetts, Milford, USA) in negative ionization mode at 0.88 kV with a 0 V sampling cone voltage and source offset voltage, 4 V trap and 2 V transfer collision energy, and 1.5 mL/min trap gas flow. The scrambling experiment between **1**_6_ and **1D**_6_ was presented as an example. Solutions of **1**_6_ and of **1D**_6_ ([GSA] = 0.8 mM) in H_2_O were prepared separately. The solutions of **1**_6_ (150 μL) and of **1D**_6_ (150 μL) were added to an Eppendorf microcentrifuge tube. Then, the concentration of GSAs was adjusted to be 0.4, 0.1, and 0.025 mM by addition of Milli-Q water. The scrambling of the GSAs was monitored by mass spectrometry. A portion of the mixture was taken. After addition of 1 mM H_2_SO_4_ aq., this solution was immediately injected into gold-coated glass capillaries made in house (~5 μL sample loaded per analysis) and the mass spectrum was recorded. The spectra were calibrated using 1 mg/mL CsI and analyzed using MassLynx software (Waters).

### Monitoring of scrambling by ^1^H NMR spectroscopy

Solutions of **1**_6_ and of **2**_6_ ([GSA] = 2 mM) in D_2_O were prepared separately. A solution of TMACl (8 mM, TMACl indicates tetramethylammonium chloride) in D_2_O (15 μL), which was used as an internal standard, was added to an Eppendorf microcentrifuge tube. Then the solutions of **1**_6_ (120 μL) and of **2**_6_ (120 μL) in D_2_O and D_2_O (345 μL) were added to the Eppendorf microcentrifuge tube to adjust the concentration of GSAs to 0.4 mM or the solutions of **1**_6_ (150 μL) and of **2**_6_ (150 μL) in D_2_O and D_2_O (285 μL) were added to the Eppendorf microcentrifuge tube to adjust the concentration of GSAs to 0.5 mM. The scrambling of the GSAs was monitored at 25 °C by ^1^H NMR spectroscopy. The existence ratios of **1**_6_ and **2**_6_ were determined by their integrated values against that of the signal of TMA^+^. Three *p*-tolyl methyl signals observed at 0.5–2 ppm were used to determine the existence ratio of **1**_6_. As **2**_6_ lacks *p*-tolyl methyl groups, *N*-methyl signals at 3–4 ppm were used to determine the existence ratio of **2**_6_. In order to confirm the reproducibility of the measurements, the same experiments were carried out three times. Similar procedures were conducted for the scrambling of the nanocubes with guest molecules. See supplementary information for more details (Supplementary Figs. 3, 4 and 7–14).

### Interconversion between ordered and disordered states in a mixture of nanocubes

An almost statistical mixture of the nanocubes **1**_*x*_**2**_6–*x*_ (*x* = 0–6) was produced from a mixture of **1**_6_ and **2**_6_ ([**1**] = [**2**] = 0.5 mM) at 25 °C for 3 days or at 50 °C for 8 h. A mixture of **1**_6_ and **2**_6_ was preferentially produced by heating at 100 °C for 20 min followed by rapid cooling at 0 °C for 20 s. The induced-sorting of **1**_6_ and **2**_6_ was monitored by variable temperature ^1^H NMR measurements (Supplementary Figs. 5 and 6).

### Kinetic lock and unlock of the induced-sorted state by the encapsulation and release of guest molecules

*n*-Hexane (Hex) (1 μL) was added via a syringe to a D_2_O solution of **1**_6_ and **2**_6_ ([**1**] = [**2**] = 0.5 mM) in an NMR tube. The suspension was mixed by shaking the NMR tube for 30 s to afford a D_2_O solution of Hex_3_@**1**_6_ and Hex_2_@**2**_6_. The encapsulated *n*-hexane was removed upon heating at 100 °C for 60 min and then a mixture of **1**_6_ and **2**_6_ was obtained by rapid cooling at 0 °C for 20 s (Supplementary Fig. 15).

## Supplementary information

Supplementary Information

Peer Review File

## Data Availability

The authors declare that all the other data supporting the findings of this study are available within the article and its supplementary information files and from the corresponding author upon request.
