# Supplementary material for: Temperature-controlled repeatable scrambling and induced-sorting of building blocks between cubic assemblies
Source: Nat Commun. 2019 Mar 29;10:1440. doi: 10.1038/s41467-019-09495-1 (PMC6441092; doi:10.1038/s41467-019-09495-1)
Supplement: Supplementary file 1 — Supplementary Information [file 41467_2019_9495_MOESM1_ESM.pdf]

# Supplementary Information

Temperature-controlled repeatable scrambling and induced-sorting of building blocks between cubic assemblies

Zhan *et al.*

## Supplementary Methods

## Synthesis of partially deuterated GSAs

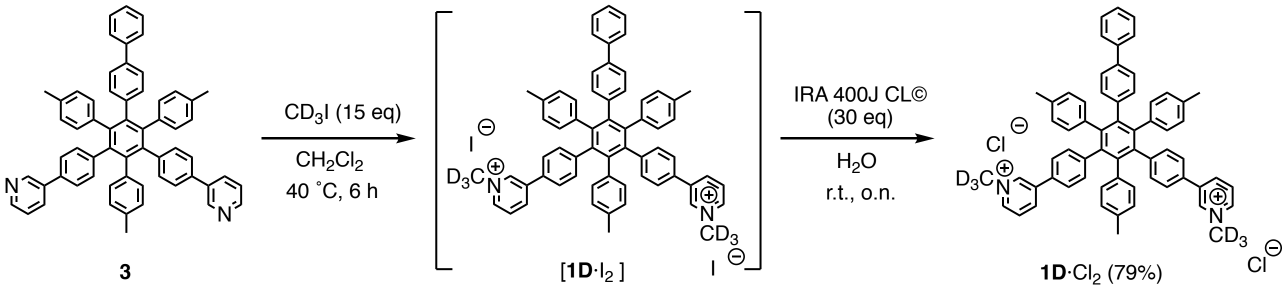


**1D**·$Cl_2$ GSA

$CD_3I$ (55 mg, 0.38 mmol) was added to a solution of compound **3** (20 mg, 0.025 mmol) in $CH_2Cl_2$ (1 mL). The reaction mixture was stirred at 40 °C for overnight under dark. Then the solvent was removed in vacuo. The crude material was washed with *n*-hexane and $CH_2Cl_2$ two times to obtain **1D**·$I_2$ (23 mg, 0.021 mmol) as a yellow solid. A suspension of **1D**·$I_2$ (23 mg, 0.021 mmol) and IRA 400J CL© (302 mg, 0.60 mmol) in water (2 mL) were stirred for overnight. Then the solution was filtered and the filtrate was concentrated in vacuo to obtain **1D**·$Cl_2$ (17 mg, 0.019 mmol) as a pale yellow solid in 79% yield in two steps. $^1$H NMR (500 MHz, $CD_3OD$, 25 °C): δ 9.12 (s, 2H), 8.74 (d, *J* = 6.0 Hz, 2H), 8.66 (d, *J* = 8.2 Hz, 2H), 8.00 (dd, *J* = 8.7, 6.1 Hz, 2H), 7.46–7.36 (m, 6H), 7.32 (t, *J* = 7.6 Hz, 2H), 7.23 (t, *J* = 7.3 Hz, 1H), 7.19–7.12 (m, 6H), 6.93 (d, *J* = 8.2 Hz, 2H), 6.88–6.80 (m, 6H), 6.73–6.65 (m, 6H), 2.04 (s, 6H), 2.01 (s, 3H); $^{13}$C NMR (125 MHz, $CD_3OD$, 298 K): δ 144.97, 144.58, 144.22, 143.30, 142.21, 142.10, 141.96, 141.79, 141.45, 140.90, 139.19, 138.67, 138.63, 136.32, 134.09, 133.02, 132.47, 132.42, 131.36, 129.67, 128.89, 128.69, 128.63, 128.08, 127.56, 126.66, 126.07, 21.03; HR-ESI-TOF-MS (*m/z*): $[M]^{2+}$ calcd. for $C_{63}H_{46}D_6N_2$, 421.2248; found, 421.2252.

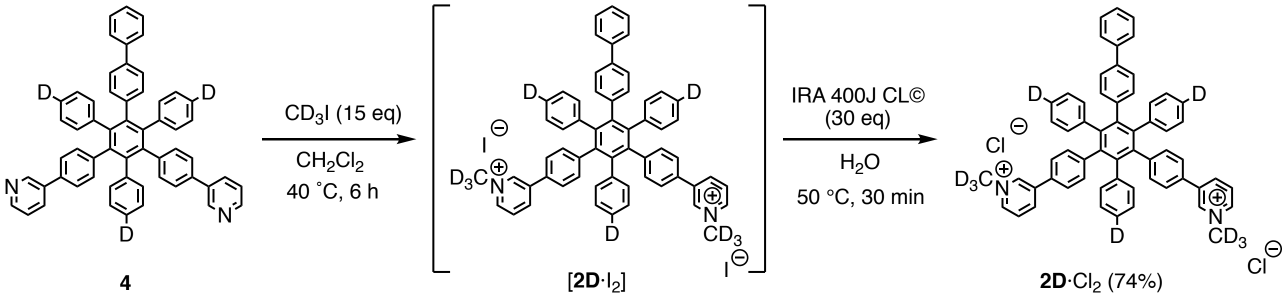


**2D**·$Cl_2$ GSA

$CD_3I$ (30 mg, 0.20 mmol) was added to a solution of compound **4** (10 mg, 0.013 mmol) in $CH_2Cl_2$ (1 mL). The reaction mixture was stirred at 40 °C for overnight under dark. Then the solvent was removed in vacuo. The crude material was washed with *n*-hexane, diethyl ether and $CHCl_3$ two times, respectively to obtain **2D**·$I_2$ (10 mg, 0.010 mmol) as a yellow solid. A suspension of **2D**·$I_2$ (10 mg, 0.010 mmol) and IRA 400J CL© (150 mg, 0.30 mmol) in water (2 mL) were stirred for 30 h at 50 °C. Then the solution was filtered and the filtrate was concentrated in vacuo to obtain **2D**·$Cl_2$ (8 mg, 0.010 mmol) as a pale yellow solid in 74% yield in two steps. $^1$H NMR (500 MHz, $CD_3OD$, 25 °C): δ 9.09 (s, 2H), 8.75 (d, *J* = 5.8 Hz, 2H), 8.63 (d, *J* = 8.4 Hz, 2H), 8.01 (dd, *J* = 8.2, 6.0 Hz, 2H), 7.44–7.36 (m, 6H), 7.32 (t, *J* = 7.5 Hz, 2H), 7.23 (t, *J* = 7.3 Hz, 1H), 7.18 (d, *J* = 8.6 Hz, 4H), 7.13 (d, *J* = 8.5 Hz, 2H), 7.02–6.94 (m, 8H), 6.90–6.85 (m, 6H); $^{13}$C NMR (125 MHz, $CD_3OD$, 298 K): δ 144.65, 144.61, 144.33, 143.32, 142.32, 141.99, 141.93, 141.76, 141.57, 141.53, 141.49, 140.81, 140.61, 139.37, 134.06, 133.00, 132.58, 132.54, 131.54, 129.67, 128.91, 128.12, 127.93, 127.88, 127.57, 126.74, 126.15. HR-ESI-TOF-MS (*m/z*): $[M]^{2+}$ calcd. for $C_{60}H_{37}D_9N_2$, 401.7107; found, 401.7090.

## The effect of guest molecules on the scrambling of GSAs between nanocubes

Scrambling experiment between $TBM_2@\mathbf{1}_6$ and $\mathbf{2}_6$: Solutions of $TBM_2@\mathbf{1}_6$ and of $\mathbf{2}_6$ ([GSA] = 2 mM) in $D_2O$ were prepared separately. A solution of TMACl (8 mM, TMACl indicates tetramethylammonium chloride) in $D_2O$ (15 μL), which was used as an internal standard, was added to an NMR tube. Then the solutions of $TBM_2@\mathbf{1}_6$ (150 μL) and of $\mathbf{2}_6$ (150 μL) and $D_2O$ (285 μL) were added to the NMR tube to adjust the concentration of GSAs to 0.5 mM. The scrambling of the GSAs was monitored at 25 °C by $^1$H NMR spectroscopy (Supplementary Figure 7).

Scrambling experiment between $\mathbf{1}_6$ and $TBM_2@\mathbf{2}_6$: Solutions of $\mathbf{1}_6$ and of $TBM_2@\mathbf{2}_6$ ([GSA] = 2 mM) in $D_2O$ were prepared separately. A solution of TMACl (8 mM, TMACl indicates tetramethylammonium chloride) in $D_2O$ (15 μL), which was used as an internal standard, was added to an NMR tube. Then the solutions of $\mathbf{1}_6$ (150 μL) and of $TBM_2@\mathbf{2}_6$ (150 μL) and $D_2O$ (285 μL) were added to the NMR tube to adjust the concentration of GSAs to 0.5 mM. The scrambling of the GSAs was monitored at 25 °C by $^1$H NMR spectroscopy (Supplementary Figure 8).

Scrambling experiment between $\mathbf{1}_6$ and $TBM_2@\mathbf{2}_6$ with the presence of insoluble TBM: Solutions of $\mathbf{1}_6$ and of $TBM_2@\mathbf{2}_6$ ([GSA] = 2 mM) in $D_2O$ were prepared separately. A solution of TMACl (8 mM) in $D_2O$ (15 μL), which was used as an internal standard, was added to an NMR tube. Then the solutions of $\mathbf{1}_6$ (150 μL) and of $TBM_2@\mathbf{2}_6$ (150 μL) and $D_2O$ (285 μL) were added to the NMR tube to adjust the concentration of GSAs to 0.5 mM. About 1 mg of TBM was added to the prepared NMR tube. The scrambling of the GSAs was monitored at 25 °C by $^1$H NMR spectroscopy (Supplementary Figure 9).

Scrambling experiment between $TBM_2@\mathbf{1}_6$ and $\mathbf{2}_6$ with the presence of insoluble TBM: Solutions of $TBM_2@\mathbf{1}_6$ and of $\mathbf{2}_6$ ([GSA] = 2 mM) in $D_2O$ were prepared separately. A solution of TMACl (8 mM) in $D_2O$ (15 μL), which was used as an internal standard, was added to an NMR tube. Then the solutions of $TBM_2@\mathbf{1}_6$ (150 μL) and of $\mathbf{2}_6$ (150 μL) and $D_2O$ (285 μL) were added to the NMR tube to adjust the concentration of GSAs to 0.5 mM. About 1 mg of TBM was added to the prepared NMR tube. The scrambling of the GSAs was monitored at 25 °C by $^1$H NMR spectroscopy (Supplementary Figure 10).

Scrambling experiment between $PCCP_2@\mathbf{1}_6$ and $\mathbf{2}_6$: Solutions of $PCCP_2@\mathbf{1}_6$ and of $\mathbf{2}_6$ ([GSA] = 2 mM) in $D_2O$ were prepared separately. A solution of TMACl (8 mM) in $D_2O$ (15 μL), which was used as an internal standard, was added to an NMR tube. Then the solutions of $PCCP_2@\mathbf{1}_6$ (150 μL) and of $\mathbf{2}_6$ (150 μL) and $D_2O$ (285 μL) were added to the NMR tube to adjust the concentration of GSAs to 0.5 mM. The scrambling of the GSAs was monitored at 25 °C by $^1$H NMR spectroscopy (Supplementary Figure 11).

Scrambling experiment between $PCCP_2@\mathbf{1}_6$ and $PCCP_2@\mathbf{2}_6$: The solutions of $PCCP_2@\mathbf{1}_6$ and of $PCCP_2@\mathbf{2}_6$ ([GSA] = 2 mM) in $D_2O$ were prepared separately. A solution of TMACl (8 mM) in $D_2O$ (15 μL), which was used as an internal standard, was added to an NMR tube. Then the solutions of $PCCP_2@\mathbf{1}_6$ (150 μL) and of $PCCP_2@\mathbf{2}_6$ (150 μL) and $D_2O$ (285 μL) were added to the NMR tube to adjust the concentration of GSAs to 0.5 mM. The scrambling of the GSAs was monitored at 25 °C by $^1$H NMR spectroscopy (Supplementary Figure 12).

Scrambling experiment between $\mathbf{1}_6$ and $\mathbf{2}_6$ in the presence of 2 eq of PCCP: Solutions of $\mathbf{1}_6$ and of $\mathbf{2}_6$ ([GSA] = 2 mM) in $D_2O$ were prepared separately. A solution of TMACl (8 mM) in $D_2O$ (15 μL), which was used as an internal standard was added to an Eppendorf microcentrifuge tube. Then the solutions of $\mathbf{1}_6$ (150 μL) and of $\mathbf{2}_6$ (150 μL) and $D_2O$ (275 μL) were added to the Eppendorf microcentrifuge tube. A solution of NaPCCP (10 mM, 10 μL) was added and the tube

was shaken for 30 s to complete the encapsulation. The scrambling of the GSAs was monitored at 25 °C by $^{1}H$ NMR spectroscopy (Supplementary Figure 13).

Scrambling experiment between $\mathbf{1}_6$ and PCCP$_2$@$\mathbf{2}_6$: The solutions of $\mathbf{1}_6$ and of PCCP$_2$@$\mathbf{2}_6$ ([GSA] = 2 mM) in $D_2O$ were prepared separately. A solution of TMACl (8 mM) in $D_2O$ (15 μL), which was used as an internal standard, was added to an Eppendorf microcentrifuge tube. Then the solutions of $\mathbf{1}_6$ (150 μL) and of PCCP$_2$@$\mathbf{2}_6$ (150 μL) and $D_2O$ (285 μL) were added to the Eppendorf microcentrifuge tube to adjust the concentration of GSAs to 0.5 mM. The scrambling of the GSAs was monitored at 25 °C by $^{1}H$ NMR spectroscopy (Supplementary Figure 14).

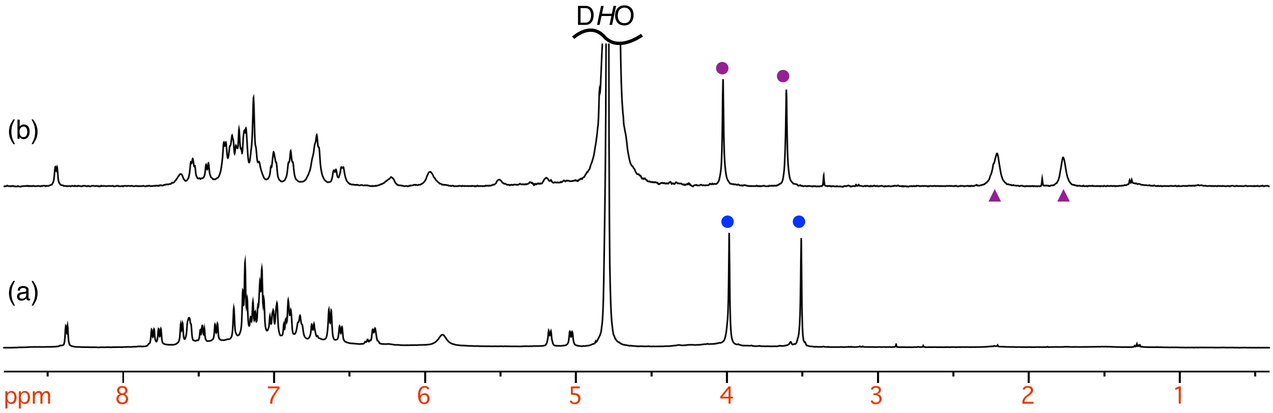


**Supplementary Figure 1** | $^{1}H$ NMR spectra of the host-guest complexes between $\mathbf{2}_6$ and *n*-hexane (500 MHz, 25 °C, $D_2O$, [**2**] = 1.0 mM). (a) $\mathbf{2}_6$. (b) Hex$_2$@$\mathbf{2}_6$. Hex indicates *n*-hexane. Blue and violet solid circles indicate the signals of the *N*-methyl signals of $\mathbf{2}_6$ and Hex$_2$@$\mathbf{2}_6$, respectively. Violet solid triangle indicates the encapsulated Hex.

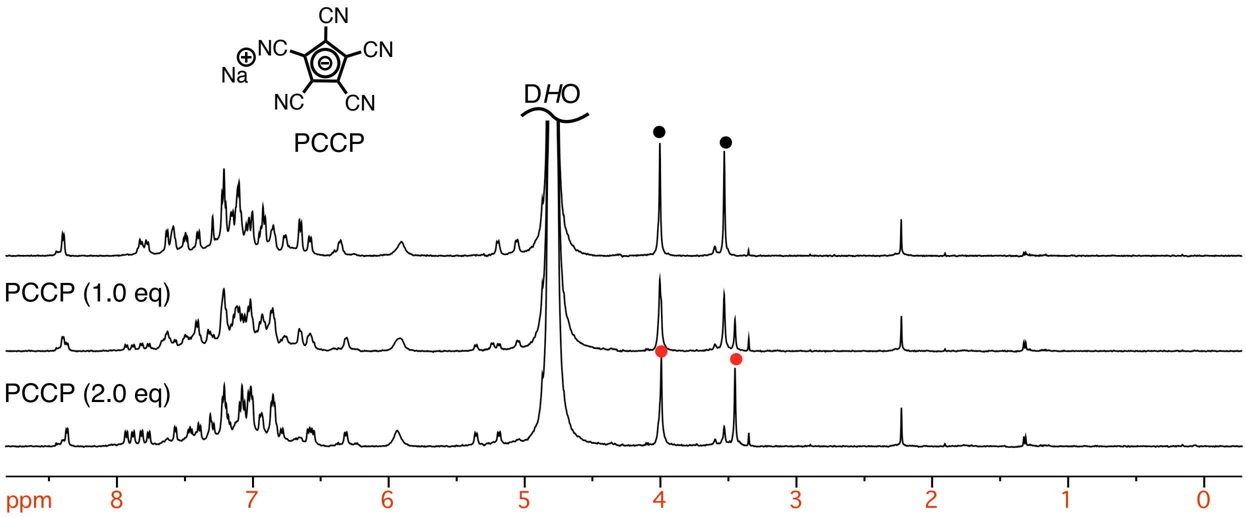


**Supplementary Figure 2** | $^{1}H$ NMR titration experiments of PCCP against $\mathbf{2}_6$ (500 MHz, 25 °C, $D_2O$, [**2**] = 1.0 mM, PCCP indicates pentacyanocyclopentadienide). Red and black solid circles indicate the signals of the *p*-tolyl methyl signals of PCCP$_2$@$\mathbf{2}_6$ and $\mathbf{2}_6$, respectively.

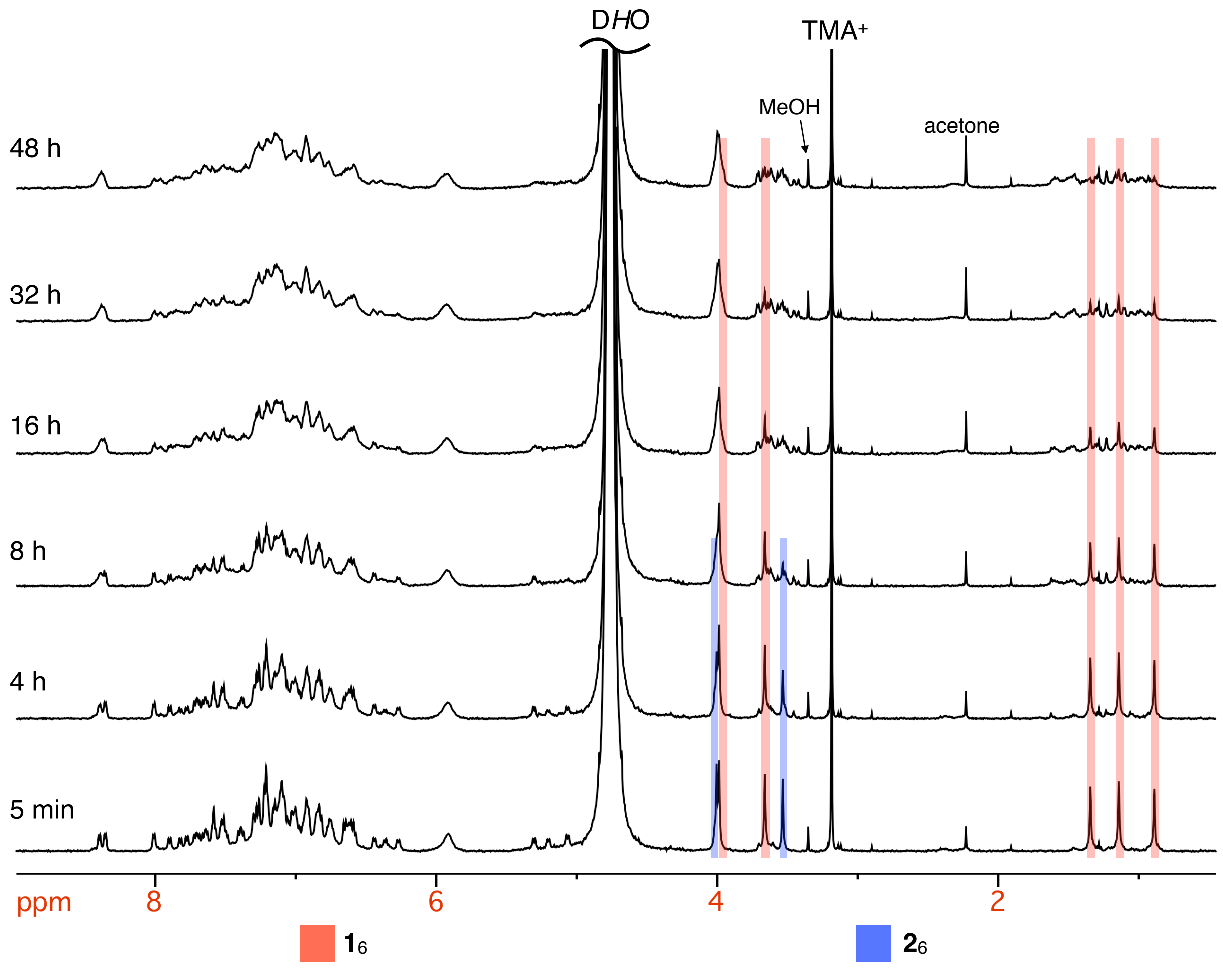


**Supplementary Figure 3 |** $^{1}$H NMR spectra of the scrambling experiment between $\mathbf{1}_6$ and $\mathbf{2}_6$ (500 MHz, 25 °C, $D_2O$, [**1**] = [**2**] = 0.4 mM). $TMA^+$ indicates tetramethylammonium used as an internal standard.

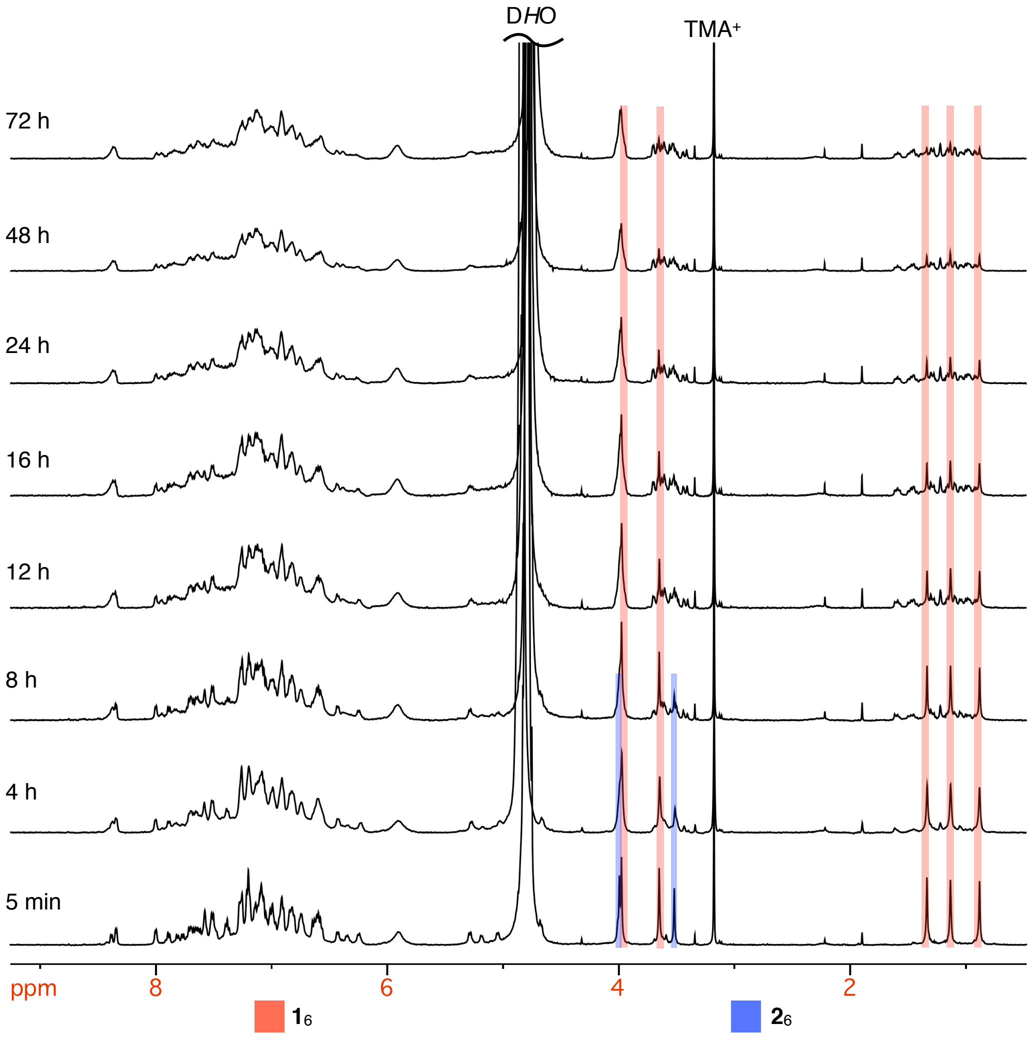


**Supplementary Figure 4 |** $^{1}$H NMR spectra of the scrambling experiment between $\mathbf{1}_6$ and $\mathbf{2}_6$ (500 MHz, 25 °C, $D_2O$, [**1**] = [**2**] = 0.5 mM). $TMA^+$ indicates tetramethylammonium used as an internal standard.

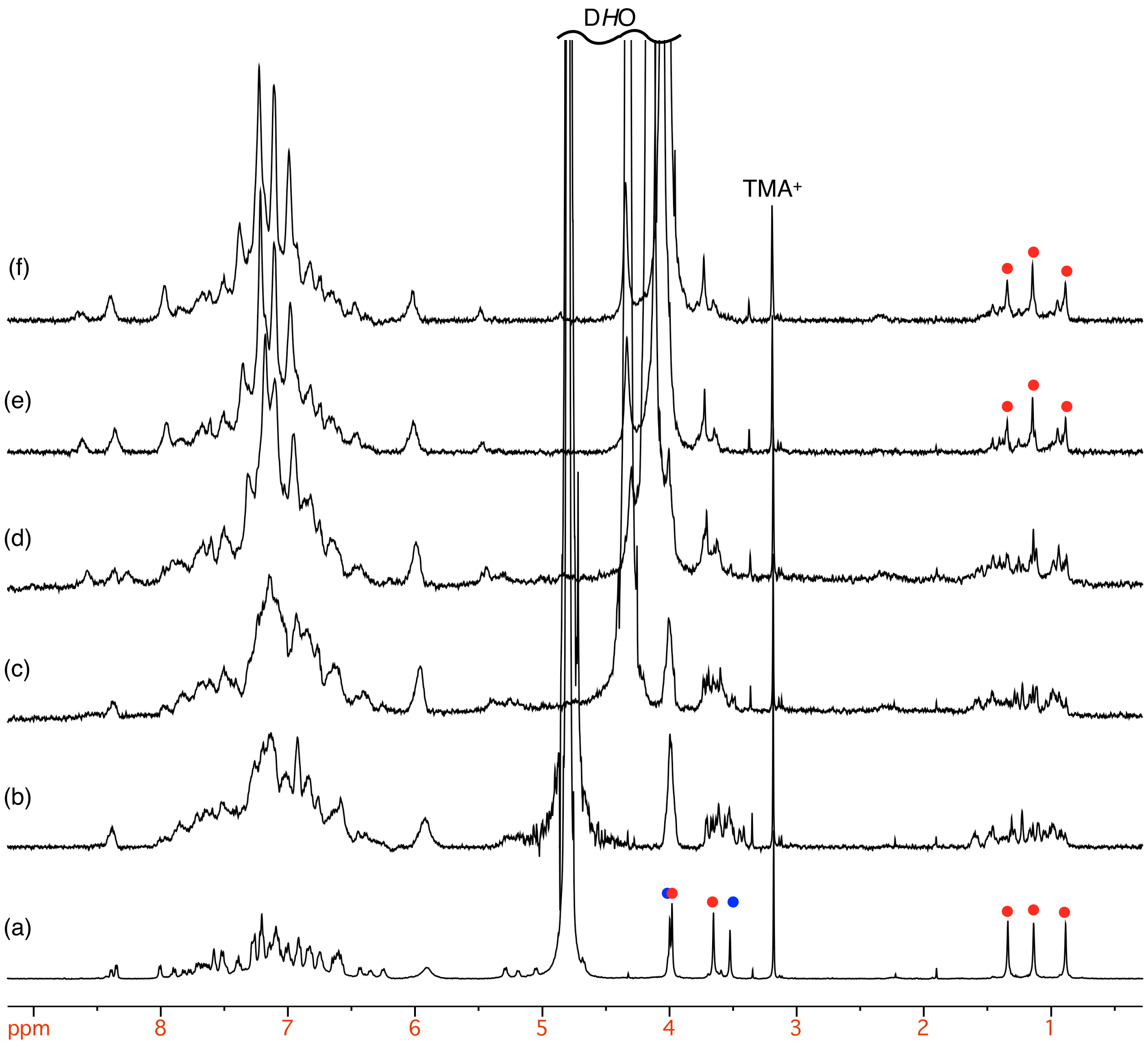


**Supplementary Figure 5 |** $^{1}$H NMR monitor of the induced-sorting of $\mathbf{1}_6$ upon heating (500 MHz, $D_2O$, [**1**] = [**2**] = 0.5 mM). (a) A mixture of $\mathbf{1}_6$ and $\mathbf{2}_6$ at 25 °C measured right after the mixing of $\mathbf{1}_6$ and $\mathbf{2}_6$. (b) An almost statistical mixture of $\mathbf{1}_x\mathbf{2}_{6-x}$ ($x$ = 0 – 6) at 25 °C (after the convergence). (c) heated at 70 °C. (d) heated at 90 °C. (e) heated at 95 °C. (f) heated at 100 °C. Red and blue solid circles indicate the signals of $\mathbf{1}_6$ and $\mathbf{2}_6$, respectively. $TMA^+$ indicates tetramethylammonium used as an internal standard.

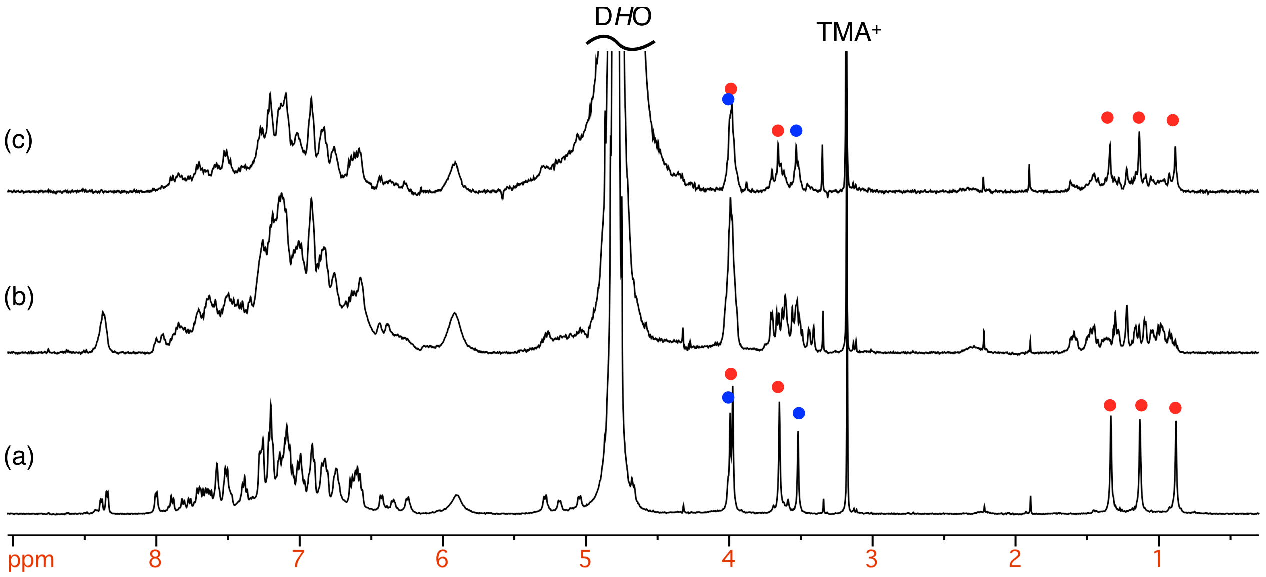


**Supplementary Figure 6 |** $^{1}$H NMR monitor of one cycle of the transitions between the ordered and disordered states by changing temperature (500 MHz, 25 °C, $D_2O$, [**1**] = [**2**] = 0.5 mM). (a) A mixture of $\mathbf{1}_6$ and $\mathbf{2}_6$ measured right after the mixing $\mathbf{1}_6$ and $\mathbf{2}_6$. (b) An almost statistical mixture of $\mathbf{1}_x\mathbf{2}_{6-x}$ ($x$ = 0 – 6) after the convergence. (c) A mixture of $\mathbf{1}_6$ and $\mathbf{2}_6$ was preferentially produced by heating at 100 °C followed by rapid cooling at 0 °C. Red and blue solid circles indicate the signals of $\mathbf{1}_6$ and $\mathbf{2}_6$, respectively. TMA$^+$ indicates tetramethylammonium used as an internal standard.

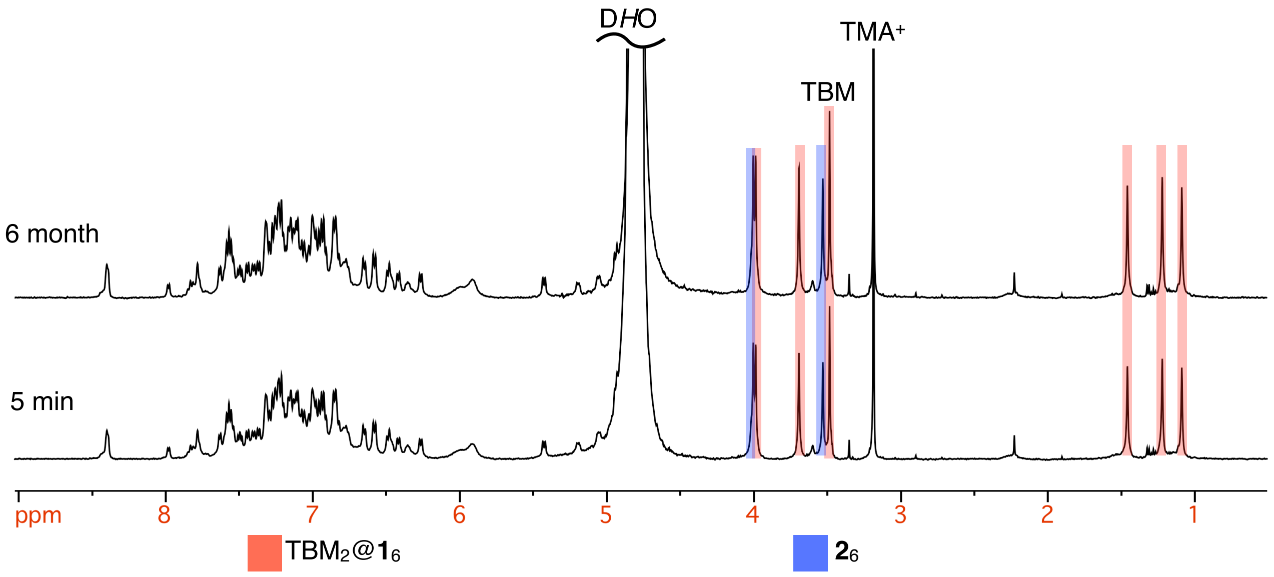


**Supplementary Figure 7 |** $^{1}$H NMR spectra of the scrambling experiment between TBM$_2$@$\mathbf{1}_6$ and $\mathbf{2}_6$ (500 MHz, 25 °C, $D_2O$, [**1**] = [**2**] = 0.5 mM). TMA$^+$ indicates tetramethylammonium used as an internal standard.

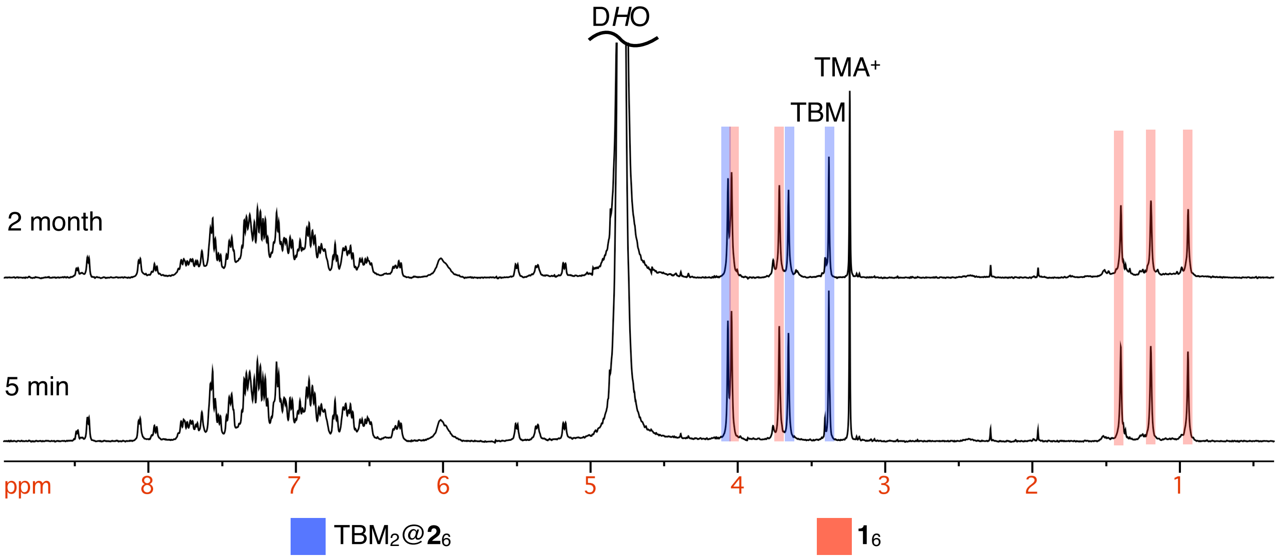


**Supplementary Figure 8 |** $^1$H NMR spectra of the scrambling experiment between $\mathbf{1}_6$ and TBM$_2$@$\mathbf{2}_6$ (500 MHz, 25 °C, $D_2O$, [**1**] = [**2**] = 0.5 mM). TMA$^+$ indicates tetramethylammonium used as an internal standard.

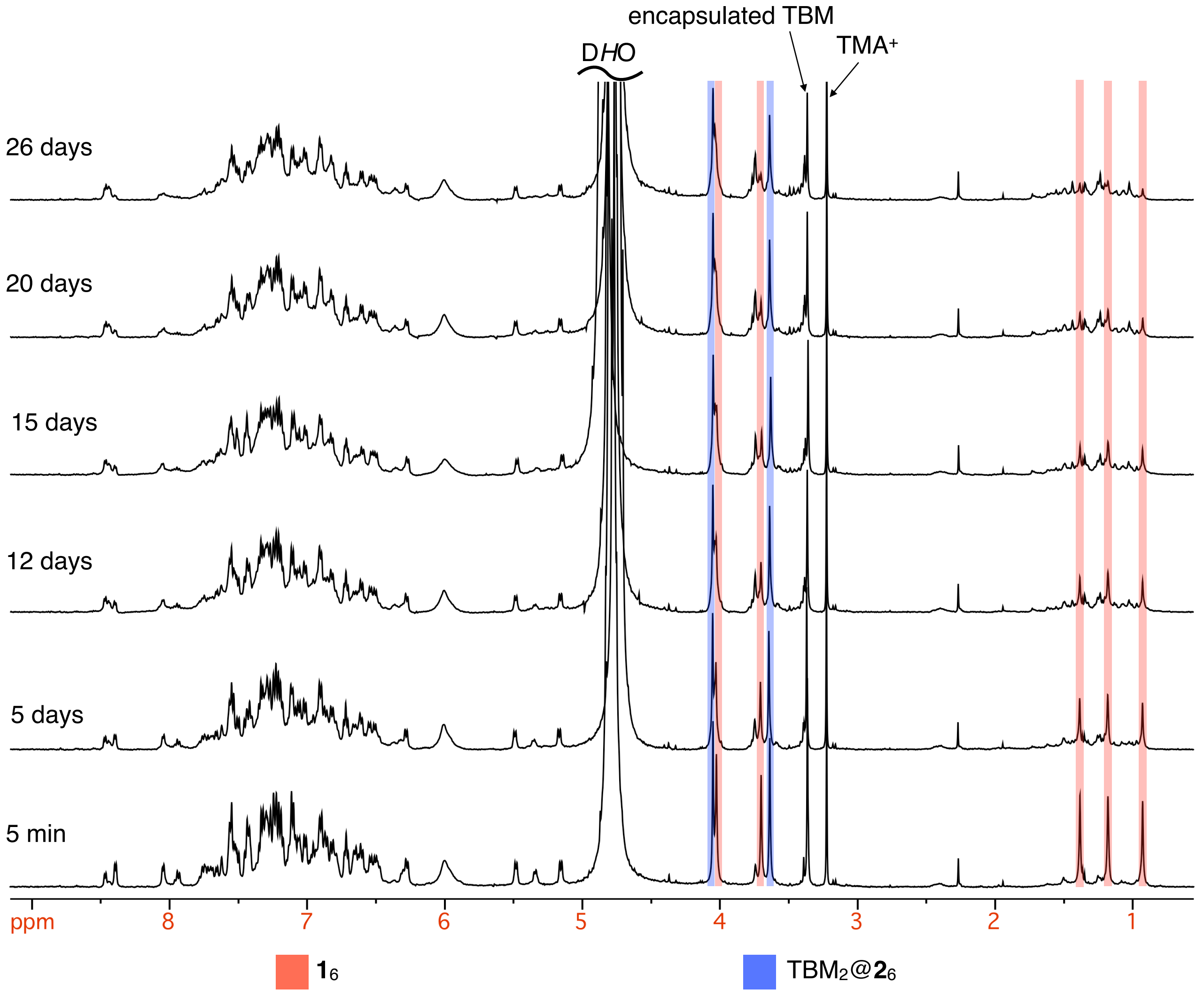


**Supplementary Figure 9 |** $^{1}$H NMR spectra of the scrambling experiment between $\mathbf{1}_6$ and $TBM_2$@$\mathbf{2}_6$ in the presence of insoluble TBM in $D_2O$ (500 MHz, 25 °C, $D_2O$, [**1**] = [**2**] = 0.5 mM). $TMA^+$ indicates tetramethylammonium used as an internal standard.

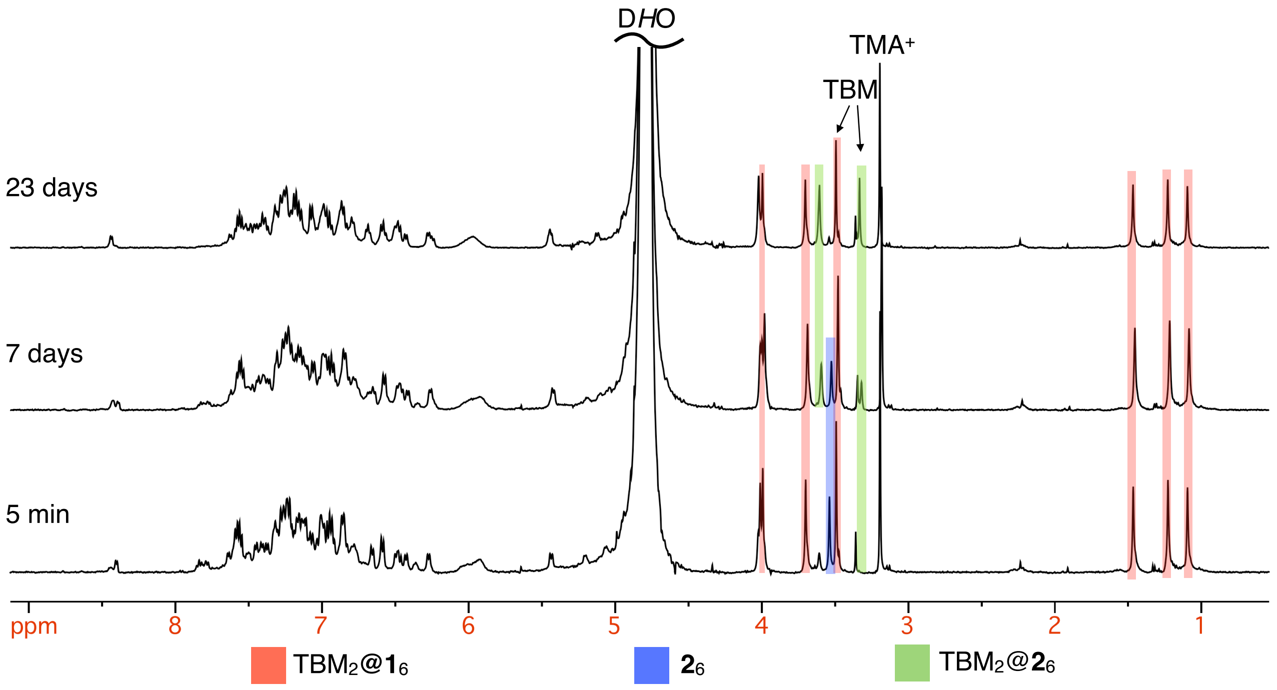


**Supplementary Figure 10 |** $^1$H NMR spectra of the scrambling experiment between $TBM_2@\mathbf{1}_6$ and $\mathbf{2}_6$ in the presence of insoluble TBM in $D_2O$ (500 MHz, 25 °C, $D_2O$, [**1**] = [**2**] = 0.5 mM). $TMA^+$ indicates tetramethylammonium used as an internal standard.

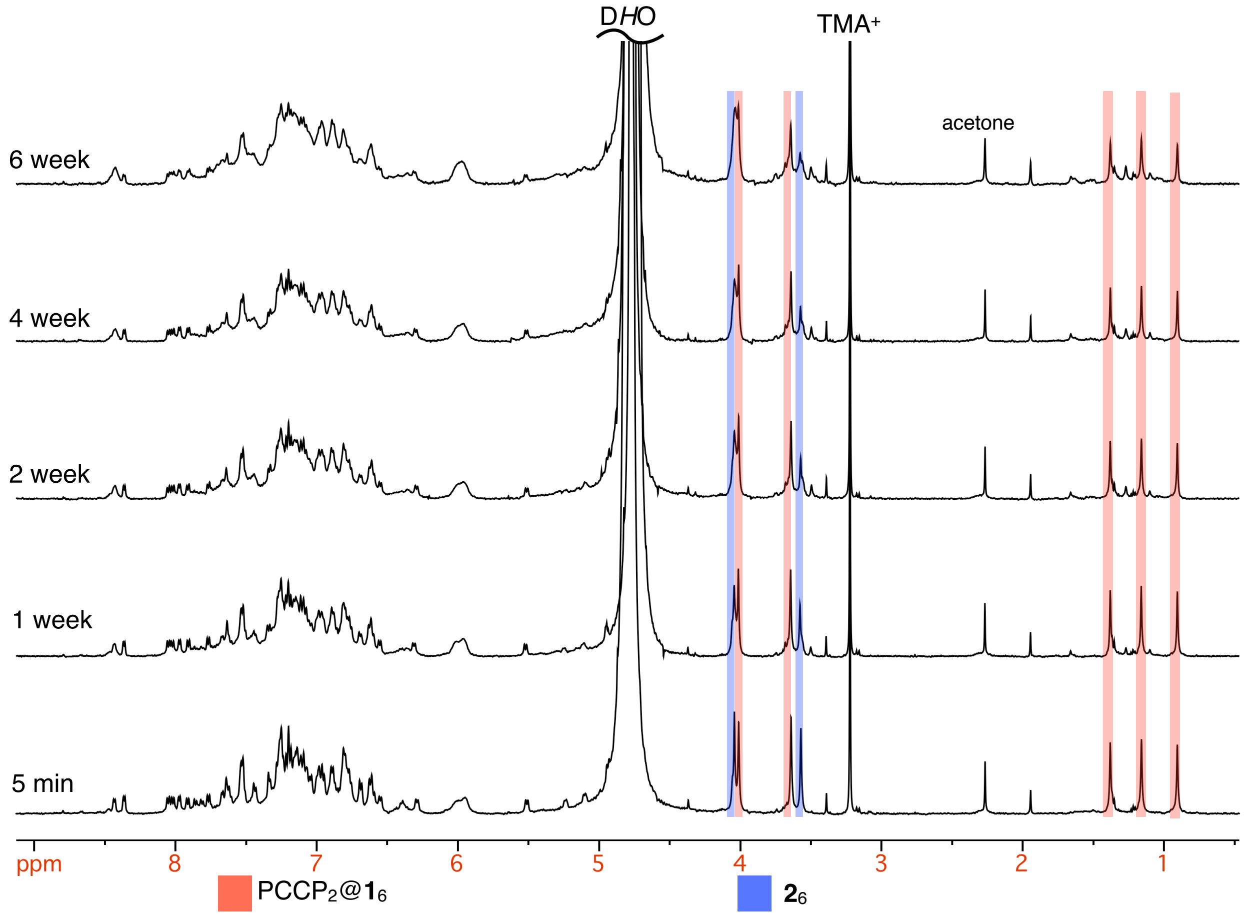


**Supplementary Figure 11 |** $^1$H NMR spectra of the scrambling experiment between $PCCP_2@\mathbf{1}_6$ and $\mathbf{2}_6$ (500 MHz, 25 °C, $D_2O$, [**1**] = [**2**] = 0.5 mM). $TMA^+$ indicates tetramethylammonium used as an internal standard.

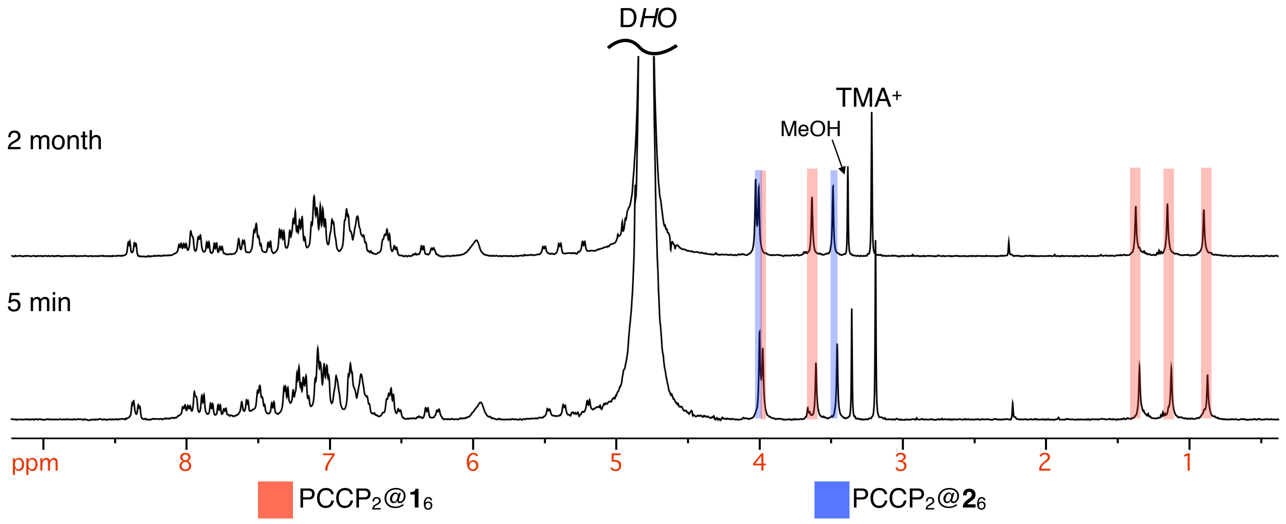


**Supplementary Figure 12 |** $^1$H NMR spectra of the scrambling experiment between PCCP$_2$@**1**$_6$ and PCCP$_2$@**2**$_6$ (500 MHz, 25 °C, $D_2O$, [**1**] = [**2**] = 0.5 mM). TMA$^+$ indicates tetramethylammonium used as an internal standard.

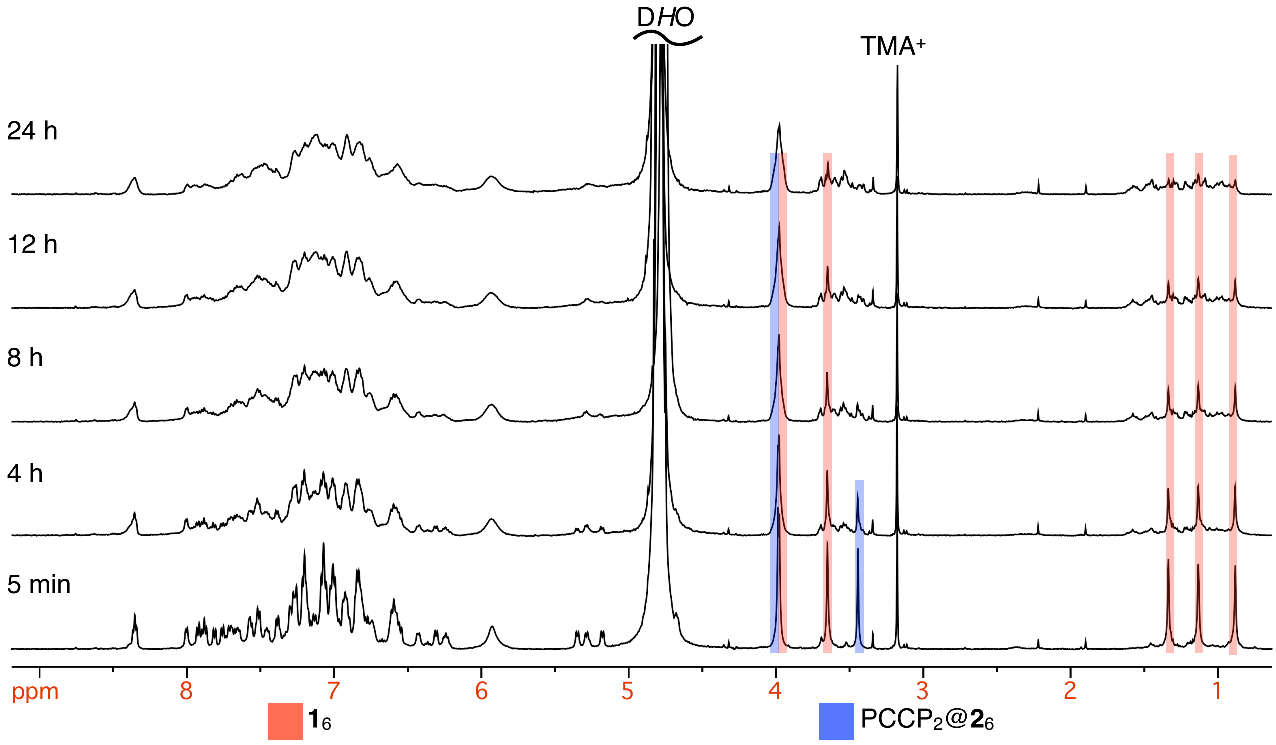


**Supplementary Figure 13 |** $^1$H NMR spectra of the scrambling experiment between **1**$_6$ and **2**$_6$ in the presence of 2 eq of PCCP (500 MHz, 25 °C, $D_2O$, [**1**] = [**2**] = 0.5 mM). TMA$^+$ indicates tetramethylammonium used as an internal standard.

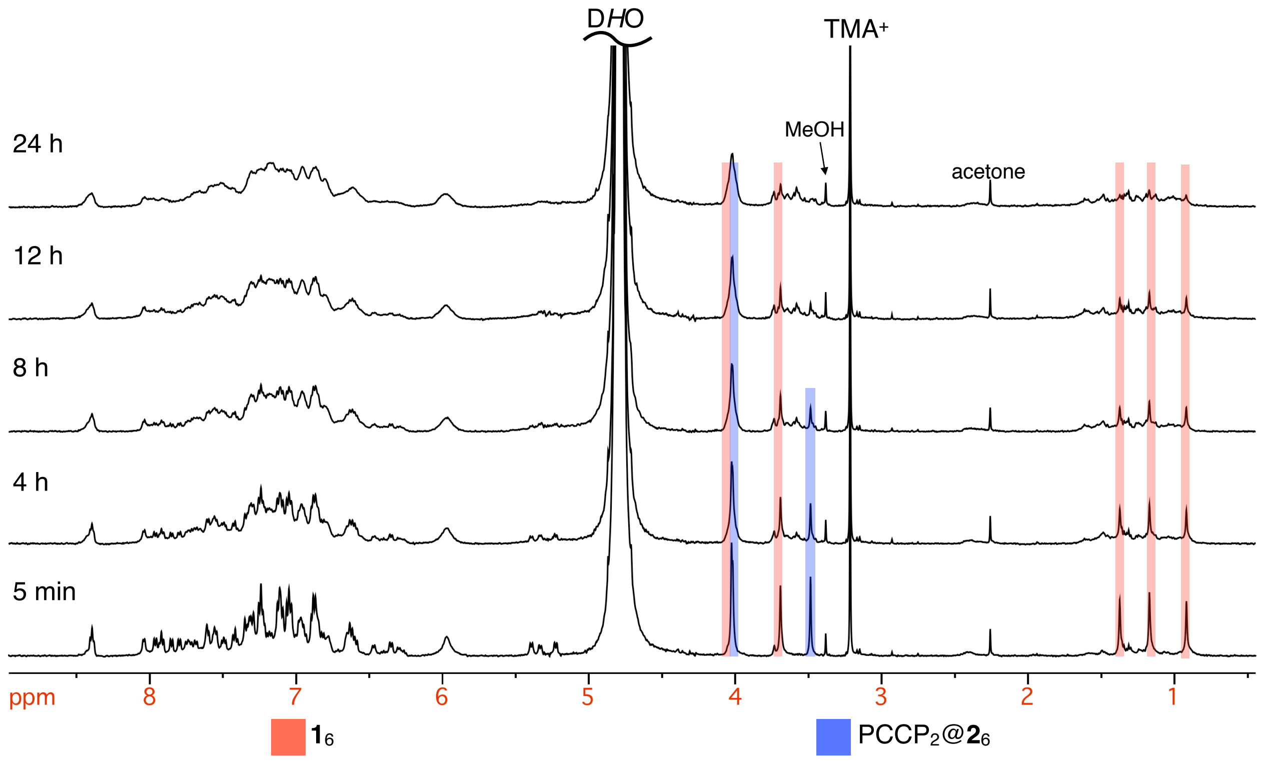


**Supplementary Figure 14 |** $^1$H NMR spectra of the scrambling experiment between $\mathbf{1}_6$ and PCCP$_2$@$\mathbf{2}_6$ (500 MHz, 25 °C, $D_2O$, [**1**] = [**2**] = 0.5 mM). TMA$^+$ indicates tetramethylammonium used as an internal standard.

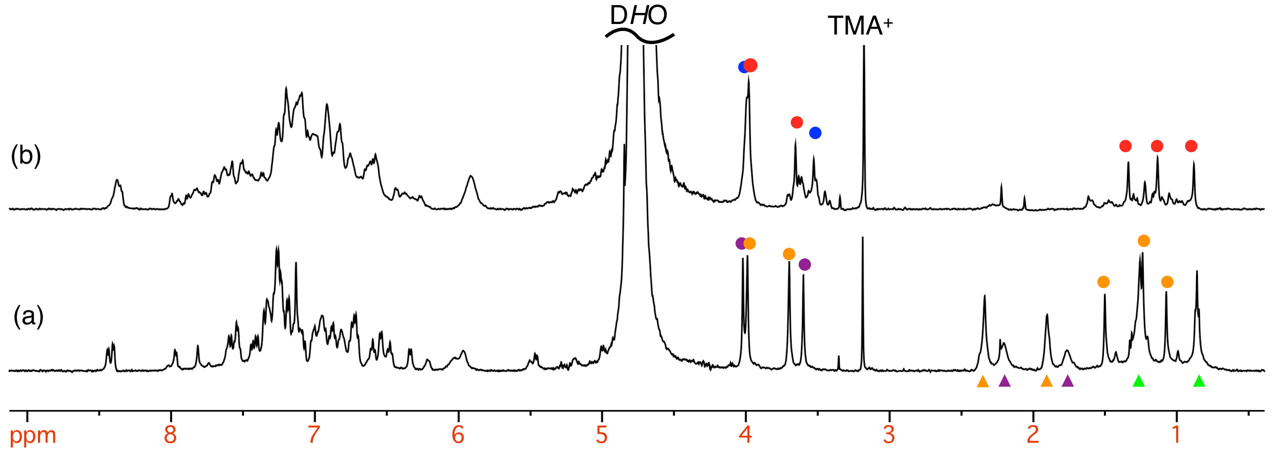


**Supplementary Figure 15 |** $^1$H NMR monitoring of kinetic lock and unlock of the induced-sorted state (500 MHz, 25 °C, $D_2O$, [**1**] = [**2**] = 0.5 mM). (a) The locked state: a mixture of Hex$_3$@$\mathbf{1}_6$ and Hex$_2$@$\mathbf{2}_6$. Hex indicates *n*-hexane. Orange and violet solid circles indicate the signals of Hex$_3$@$\mathbf{1}_6$ and Hex$_2$@$\mathbf{2}_6$, respectively. Orange, violet and green solid triangles indicate the signals of Hex encapsulated in $\mathbf{1}_6$, Hex encapsulated in $\mathbf{2}_6$ and the water-soluble free Hex, respectively. (b) The unlocked state: a mixture of $\mathbf{1}_6$ and $\mathbf{2}_6$. The encapsulated Hex was removed upon heating at 100 °C. Red and blue solid circles indicate the signals of $\mathbf{1}_6$ and $\mathbf{2}_6$, respectively. TMA$^+$ indicates tetramethylammonium used as an internal standard.

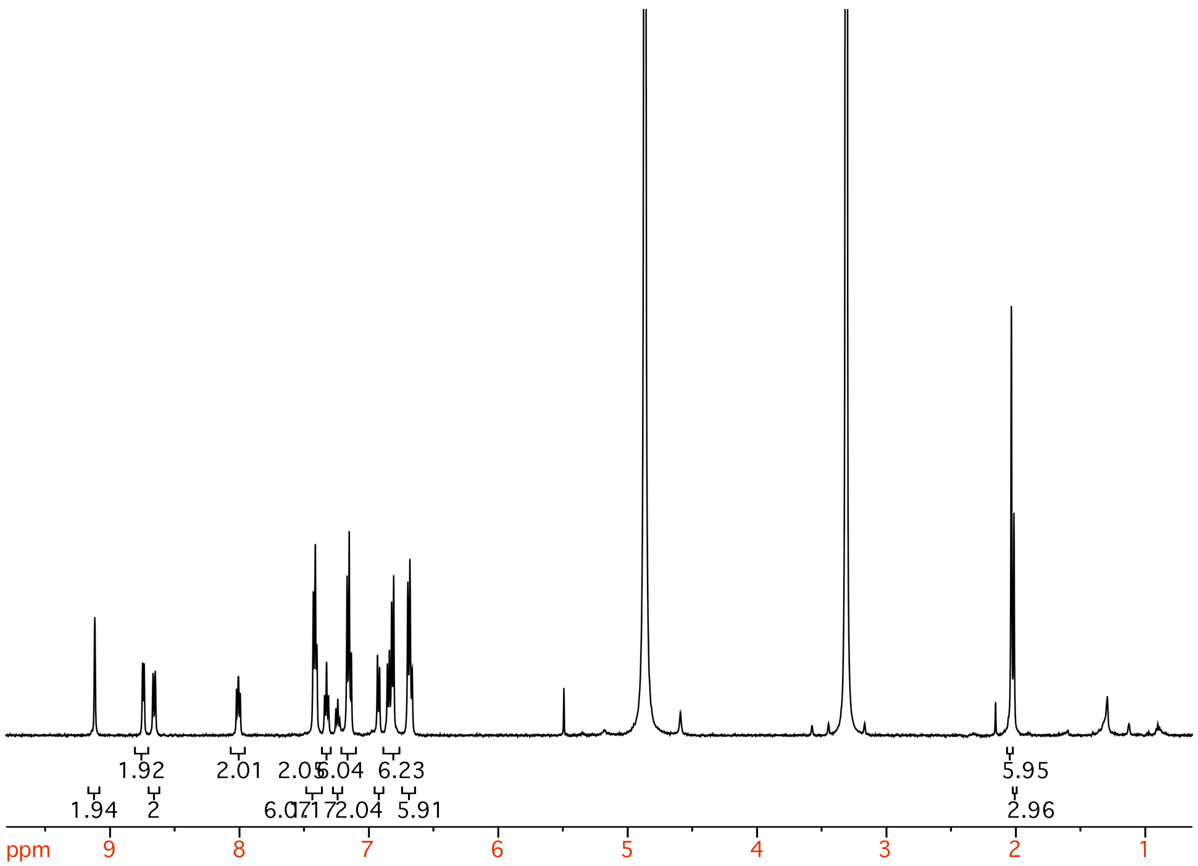


**Supplementary Figure 16 |** $^{1}$H NMR spectrum (500 MHz, $CD_3OD$, 25 °C) of **1D**·$Cl_2$.

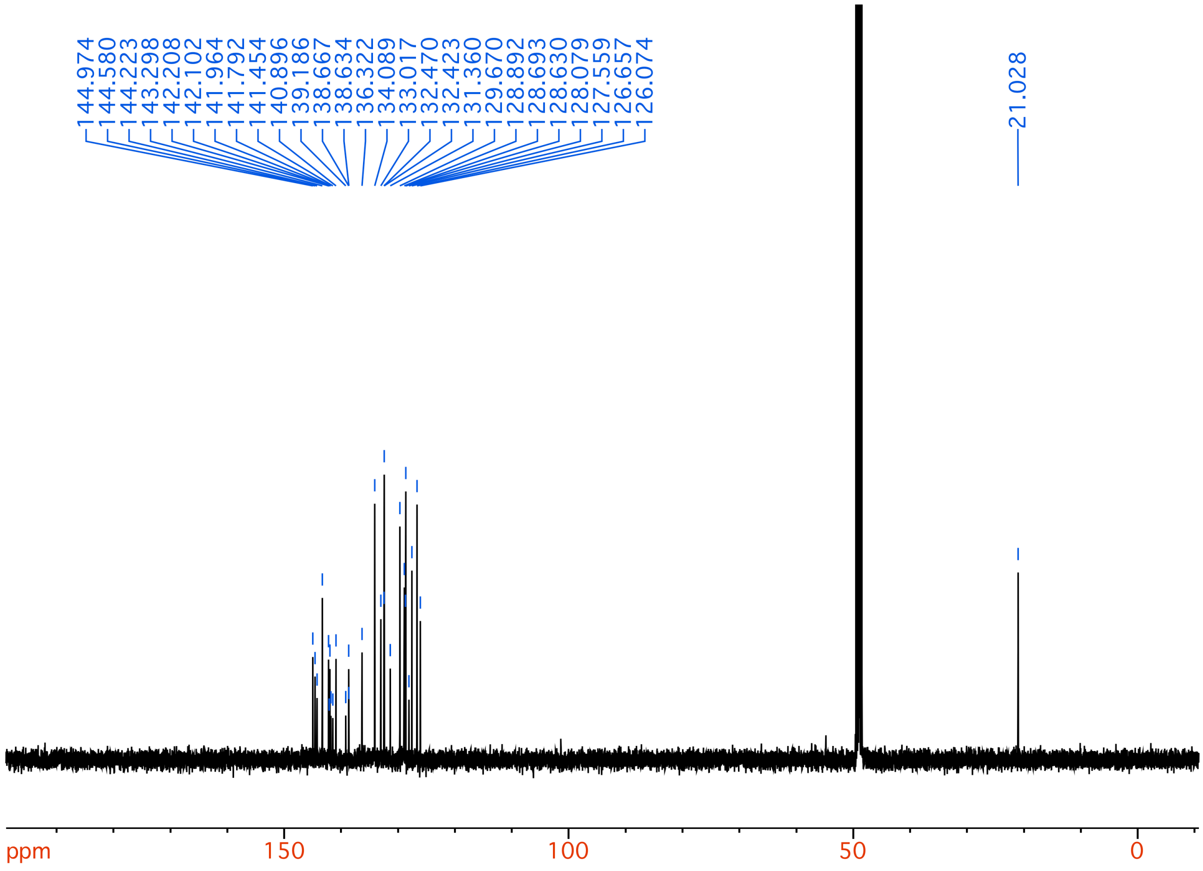


**Supplementary Figure 17 |** $^{13}$C NMR spectrum (125 MHz, $CD_3OD$, 25 °C) of **1D**·$Cl_2$

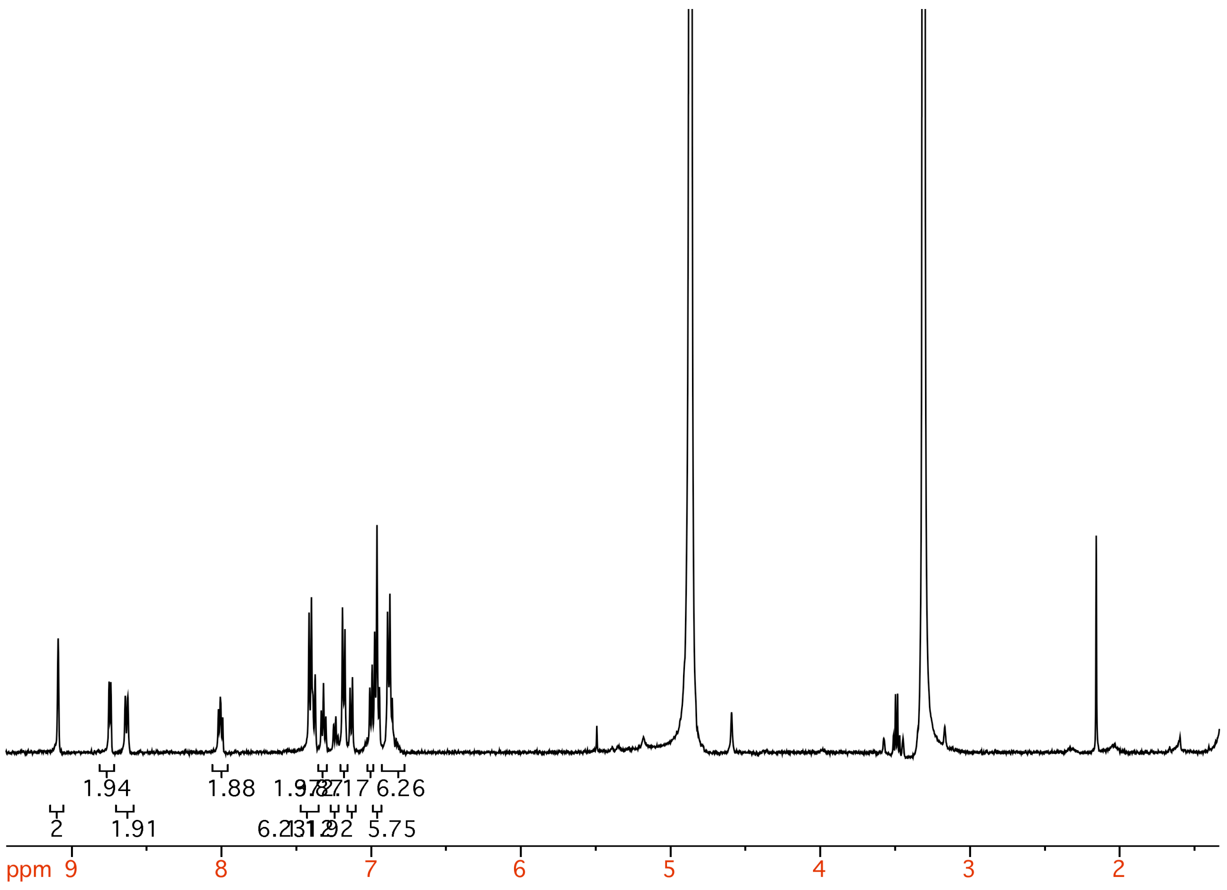


**Supplementary Figure 18 |** $^{1}H$ NMR spectrum (500 MHz, $CD_3OD$, 25 °C) of **2D**·$Cl_2$.

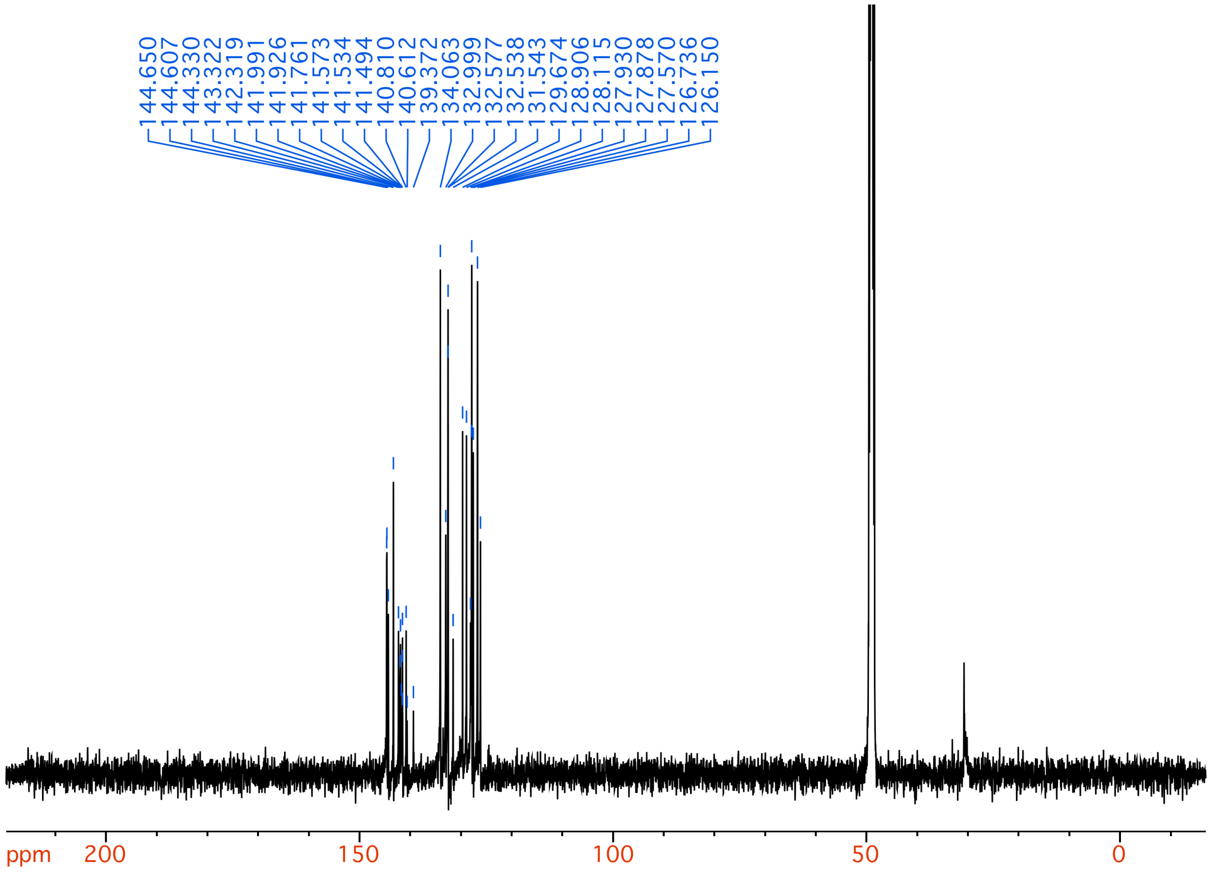


**Supplementary Figure 19 |** $^{13}C$ NMR spectrum (125 MHz, $CD_3OD$, 25 °C) of **2D**·$Cl_2$
