# Supplementary material for: Temperature-controlled repeatable scrambling and induced-sorting of building blocks between cubic assemblies
Source: Nat Commun. 2019 Mar 29;10:1440. doi: 10.1038/s41467-019-09495-1 (PMC6441092; doi:10.1038/s41467-019-09495-1)
Supplement: Supplementary file 2 — Peer Review File [file 41467_2019_9495_MOESM2_ESM.pdf]

Reviewers' comments:

Reviewer #1 (Remarks to the Author):

Review Report for:
Temperature-controlled repeatable scrambling and self-sorting of building blocks between cubic assemblies

In the manuscript, Hiraoka and coworkers describe the self-assembly of amphiphiles into cubic assemblies. When different types of building blocks are mixed, that each individually forms cubes, the resulting architecture is a statistical mixture of cubes of pure building block 1, pure building block 2 and cubes with mixtures of each building block. Because each cube (with a differing ratio of building block 1 and 2) has a slightly different melting temperature, the authors can play tricks with a melting curve to favor non-statistically expected mixtures. For example, when the cubes are heated to 100C, only cubes of pure 2 are favored. Then, when the mixture is rapidly cooled, the mixture is kinetically locked and does not scramble to form a statistical mixture, but the authors found cubes of pure 1 and pure 2. In other words, the authors navigated the energy landscape to form kinetically trapped state that resulted in pure sorted assemblies. Whether this is self-sorting (i.e., the system sorted itself) or induced sorting by navigating the landscape should be discussed.

The work is carried out carefully and relies on NMR and mass spectroscopy for the characterization of the library of the self-assembled states. The work is topical, to the point, and relevant for the field. I thus encourage publication in Nature Communications.

Some minor points have arisen when reading the manuscript:

I believe the free energy landscape in figure 1a helps the reader to understand the system. Perhaps another landscape would help the reader understand the role of the guests in Figure 4a.
While the text is generally well-written, the figure captions need some work. For example, Figure 2a-c does not describe the actual data (what are we actually looking at), merely the conclusion of the data.

Reference 29 and 31 are the same. Recent work form that groups uses non-equilibrium to access self-selection: Nature Communications, volume 9, Article number: 2044 (2018)

Reviewer #2 (Remarks to the Author):

The authors of the paper under review present a very non-intuitive way of influencing the composition of a complex dynamic system consisting of self-assembled cages. They used differences in thermal stability of the cages to control thermodynamic equilibrium and have shown that one type of the cages can be thermally amplified at the expense of other species . At room temperature the two types of monomers form statistically distributed heteroleptic and homoleptic hexameric cages composed of both components. Upon rising of the temperature one of type of the homoleptic cages gets disintegrated that leads to the shift of the thermodynamic equilibrium, rearrangement of the heteroleptic cages and, as a consequence, amplification of the second type of a homoleptic cage. I think that the concept is ingenious, although not very general. Despite the fact that I really value this approach and do not question the final conclusion, I think this work has many experimental deficiencies and requires substantial re-writing to make it scientifically sound. Therefore, I do not recommend publication of this paper in Nature Communications in the current form. Below I specify the reasons and suggested corrections.

1. The wording is misleading in this paper. The authors claim transformation between “ordered and disordered state controlled by temperature”, while the transformation involves disintegration of one type of the capsules into monomers, therefore, this statement is not true.

2. The introduction sounds as if the authors have discovered a new phenomenon that stands in contrary to the general rules of thermodynamics, while, of cause, nothing is against the rules. The authors just presented the situation from the point of view of single species (that get ordered) but not from the view of the whole system (that gets disordered). I am not saying that their approach is not interesting. It just should not be advertised in that way, because it is a scientific paper after all.
3. The authors analyze really complex mixture and severely overlapped NMR spectra. Therefore it is difficult to interpret the amounts of the homoleptic species in a quantitative way, considering that many species may also have broad signals in this region and the main integration component can come from the broad “hill” rather than from the main sharp signal. Integration in such an overlapped region also depends crucially on the integration width. Therefore, the percentage amounts of the ordered state are rather roughly determined. The authors should elaborate reliable, repeatable way of integration and estimate the errors.

4. From the presented data it is also not possible to say how the authors determined the moment when they consider that equilibrium was reached – by the end of visible changes ? (which is quite imprecise) or by some numerical method (what was he threshold) ? The time of reaching the equilibrium is discussed in many places and the influence of many factors on this value is discussed. Therefore, it is crucial to determine these values in a repeatable way.

5. Equilibration processes are extremely sensitive to concentration, temperature and additives. The authors did not specify how the temperature was controlled between NMR measurements. Together with imprecise determination of the equilibration timing, these two factors makes the quantitative conclusions very vague.

6. In the discussion on the influence of added guests on the equilibration process the authors completely ignored the solubility of the guests. The reader can guess that guest TBM is completely insoluble in water, so the equilibration proceeds at the liquid/solid interface, guest PCCP can be slightly soluble in water (although nothing is said about it), while hexane is immiscible in water so equilibration proceeds at the liquid/liquid interface. Therefore, there are three completely different situations with completely different rate limiting factors (phase transfer or even grain size and stirring speed). The authors take these factors into account experimentally and also consider them in the discussion.

7. Considering all these reservation the conclusions should be much more balanced.
8. The paper also needs language corrections for improvement of the clarity and general correctness.

Reviewer #3 (Remarks to the Author):

This is an interesting paper in which the scrambling of two self-assembled cubic structures can be controlled through a variety of means--temperature, or neutral and anionic guests. The work is thoroughly done, but I do not think it has the novelty to warrant publication in Nature Communications. It is the fourth paper in the series by the authors, detailing further investigations of the systems that were already published and shown to encapsulate guests. In addition, similar guest-modulated transformations have been precedented by e.g. Nitschke, so the phenomenon is not

entirely new either. This work is solid, but should be published in a lower-profile journal.

Response to Reviewers for NCOMMS-18-32696-T

To reviewer 1

We are grateful for your careful review and valuable comments. Our response is as follows. Thank you again for your time in advance.

Reviewer 1's comments and our response

Comment:

In the manuscript, Hiraoka and coworkers describe the self-assembly of amphiphiles into cubic assemblies. When different types of building blocks are mixed, that each individually forms cubes, the resulting architecture is a statistical mixture of cubes of pure building block **1**, pure building block **2** and cubes with mixtures of each building block. Because each cube (with a differing ratio of building block **1** and **2**) has a slightly different melting temperature, the authors can play tricks with a melting curve to favor non-statistically expected mixtures. For example, when the cubes are heated to 100 °C, only cubes of pure **2** are favored. Then, when the mixture is rapidly cooled, the mixture is kinetically locked and does not scramble to form a statistical mixture, but the authors found cubes of pure **1** and pure **2**. In other words, the authors navigated the energy landscape to form kinetically trapped state that resulted in pure sorted assemblies. *1Whether this is self-sorting (i.e., the system sorted itself) or induced sorting by navigating the landscape should be discussed.*

The work is carried out carefully and relies on NMR and mass spectroscopy for the characterization of the library of the self-assembled states. The work is topical, to the point, and relevant for the field. I thus encourage publication in *Nature Communications*.

Some minor points have arisen when reading the manuscript:

2I believe the free energy landscape in figure 1a helps the reader to understand the system. Perhaps another landscape would help the reader understand the role of the guests in Figure 4a.

3While the text is generally well-written, the figure captions need some work. For example, Figure 2a-c does not describe the actual data (what are we actually looking at), merely the conclusion of the data.

4Reference 29 and 31 are the same. Recent work form that groups uses non-equilibrium to access self-selection: Nature Communications, volume 9, Article number: 2044 (2018)

Comment 1:

Whether this is self-sorting (i.e., the system sorted itself) or induced sorting by navigating the landscape should be discussed.

Response to the reviewer

We really appreciate such a valuable question of this reviewer's. Generally, self-sorting is relevant to molecular self-assembly. In the case of the self-assembly of the nanocubes from two kinds of gear-shaped amphiphiles (GSAs), **1** and **2**, if we define narcissistic self-sorting as the selective formation of $\mathbf{1}_6$ and $\mathbf{2}_6$, the phenomenon we observed is not strict self-sorting because $\mathbf{1}_6$ was selectively formed at 100 °C, while $\mathbf{2}_6$ was disassembled into monomer **2**. As to the point whether this phenomenon took place spontaneously or not, considering the fact that a mixture of $\mathbf{1}_6$ and monomer **2** was produced upon heating, induced-sorting is better to describe this observation. Thus, the title of this paper is slightly changed to indicate induced-sorting as follows:

The revised title of this paper: Temperature-controlled repeatable scrambling and induced-sorting of building blocks between cubic assemblies

The words "self-sorting" in main text were also replaced with "induced-sorting".

Comment 2:
I believe the free energy landscape in figure 1a helps the reader to understand the system. Perhaps another landscape would help the reader understand the role of the guests in Figure 4a.

Response to the reviewer
According to the reviewer's advice, Figure 4d to explain the role of the guest molecule(s) encapsulated in Figure 4a is added in revised Figure 4.

Comment 3:
While the text is generally well-written, the figure captions need some work. For example, Figure 2a-c does not describe the actual data (what are we actually looking at), merely the conclusion of the data.

Response to the reviewer
According to the reviewer's suggestion, we edited the captions in the figures with additional experimental information.

Comment 4:
Reference 29 and 31 are the same. Recent work form that groups uses non-equilibrium to access self-selection: Nature Communications, volume 9, Article number: 2044 (2018)

Response to the reviewer
As the reviewer pointed out, the references 29 and 31 were the same in our previous manuscript. The correct reference is added in reference 31. The reference the reviewer kindly proposed is added as reference 34 in the revised manuscript.

To reviewer 2

We are grateful for your careful review and valuable comments. Our response is as follows. Thank you again for your time in advance.

Reviewer 2's comments and our response

Reviewer's Comment

The authors of the paper under review present a very non-intuitive way of influencing the composition of a complex dynamic system consisting of self-assembled cages. They used differences in thermal stability of the cages to control thermodynamic equilibrium and have shown that one type of the cages can be thermally amplified at the expense of other species. At room temperature the two types of monomers form statistically distributed heteroleptic and homoleptic hexameric cages composed of both components. Upon rising of the temperature one of type of the homoleptic cages gets disintegrated that leads to the shift of the thermodynamic equilibrium, rearrangement of the heteroleptic cages and, as a consequence, amplification of the second type of a homoleptic cage. I think that the concept is ingenious, although not very general. Despite the fact that I really value this approach and do not question the final conclusion, I think this work has many experimental deficiencies and requires substantial re-writing to make it scientifically sound. Therefore, I do not recommend publication of this paper in *Nature Communications* in the current form. Below I specify the reasons and suggested corrections.

1. The wording is misleading in this paper. The authors claim transformation between "ordered and disordered state controlled by temperature", while the transformation involves disintegration of one type of the capsules into monomers, therefore, this statement is not true.
2. The introduction sounds as if the authors have discovered a new phenomenon that stands in contrary to the general rules of thermodynamics, while, of cause, nothing is against the rules. The authors just presented the situation from the point of view of single species (that get ordered) but not from the view of the whole system (that gets disordered). I am not saying that their approach is not interesting. It just should not be advertised in that way, because it is a scientific paper after all.
3. The authors analyze really complex mixture and severely overlapped NMR spectra. Therefore, it is difficult to interpret the amounts of the homoleptic species in a quantitative way, considering that many species may also have broad signals in this region and the main integration component can come from the broad "hill" rather than from the main sharp signal. Integration in such an overlapped region also depends crucially on the integration width. Therefore, the percentage amounts of the ordered state are rather roughly determined. The authors should elaborate reliable, repeatable way of integration and estimate the errors.
4. From the presented data it is also not possible to say how the authors determined the moment when they consider that equilibrium was reached – by the end of visible changes ? (which is quite imprecise) or by some numerical method (what was he threshold) ? The time of reaching the equilibrium is discussed in many places and the influence of many factors on this value is discussed. Therefore, it is crucial to determine these values in a repeatable way.
5. Equilibration processes are extremely sensitive to concentration, temperature and additives. The authors did not specify how the temperature was controlled between NMR measurements. Together with imprecise determination of the equilibration timing, these two factors makes the quantitative conclusions very vague.

6. In the discussion on the influence of added guests on the equilibration process the authors completely ignored the solubility of the guests. The reader can guess that guest TBM is completely insoluble in water, so the equilibration proceeds at the liquid/solid interface, guest PCCP can be slightly soluble in water (although nothing is said about it), while hexane is immiscible in water so equilibration proceeds at the liquid/liquid interface. Therefore, there are three completely different situations with completely different rate limiting factors (phase transfer or even grain size and stirring speed). The authors take these factors into account experimentally and also consider them in the discussion.
7. Considering all these reservation the conclusions should be much more balanced.
8. The paper also needs language corrections for improvement of the clarity and general correctness.

Comment 1:
The wording is misleading in this paper. The authors claim transformation between "ordered and disordered state controlled by temperature", while the transformation involves disintegration of one type of the capsules into monomers, therefore, this statement is not true.

Response to the reviewer
As the reviewer pointed out, the sorted state where $\mathbf{1}_6$ and monomer **2** are produced at 100 °C is not the ordered state. The "ordered state" in this manuscript indicates a mixture of $\mathbf{1}_6$ and $\mathbf{2}_6$ produced by rapid cooling of a mixture of $\mathbf{1}_6$ and monomer **2**. In other words, the transformation from the disordered state (almost statistical mixture of nanocubes with different composition) to the ordered state where $\mathbf{1}_6$ and $\mathbf{2}_6$ mainly exist takes place by heating at 100 °C followed by rapid cooling at 0 °C. Thus, the transition between the ordered and disordered states is possible by changing the temperature (25 (or 50), 100, and 0 °C), even if the transition from the disordered state to the ordered state takes place through the transient formation of a mixture of $\mathbf{1}_6$ and monomer **2**. We carefully checked the corresponding descriptions and confirmed that these are correctly described. Followings are the descriptions concerning the transition of almost statistical mixture to a mixture of homoleptic nanocubes.

In abstract
"while homoleptic assemblies composed of single components are preferentially produced *at higher temperature (100 °C) followed by rapid cooling*."

In the introduction section
"Then, a metastable ordered state (a mixture of homoleptic nanocubes composed of a single component, A_6 and B_6) is spontaneously recovered *by heating at 100 °C and subsequent rapid cooling*"

In the conclusion section
"The result that the self-sorted state was preferred *by heating at 100 °C and subsequent rapid cooling* was realized because only the nanocubes composed of more **1** can survive at this temperature."

Comment 2:
The introduction sounds as if the authors have discovered a new phenomenon that stands in contrary to the general rules of thermodynamics, while, of cause, nothing is against the rules. The authors just presented the situation from the point of view of single species (that get ordered) but

not from the view of the whole system (that gets disordered). I am not saying that their approach is not interesting. It just should not be advertised in that way, because it is a scientific paper after all.

Response to the reviewer
As the reviewer pointed out, all the phenomena observed in this paper are clearly explained by the thermodynamic laws. The sentences in the introduction section are revised as follows:

In the introduction section
"Then, a metastable ordered state (a mixture of homoleptic nanocubes composed of a single component, A_6 and B_6) is spontaneously recovered by heating at 100 °C and subsequent rapid cooling."

Comment 3:
The authors analyze really complex mixture and severely overlapped NMR spectra. Therefore it is difficult to interpret the amounts of the homoleptic species in a quantitative way, considering that many species may also have broad signals in this region and the main integration component can come from the broad "hill" rather than from the main sharp signal. Integration in such an overlapped region also depends crucially on the integration width. Therefore, the percentage amounts of the ordered state are rather roughly determined. The authors should elaborate reliable, repeatable way of integration and estimate the errors.

Response to the reviewer
As the reviewer pointed out, the integration of the ordered state is difficult due to many complicated broad "hill" signals. To circumvent this problem, tetramethylammonium (TMA^+) chloride was added in the scrambling mixture as the internal standard (supplementary Figures 3 to 13). The existence ratios of homoleptic nanocubes in the ordered state were determined by the comparison of the integral of the main sharp signals derived from homoleptic nanocubes with that of TMA^+ without using any broadened signals of heteroleptic nanocubes to improve accuracy. The existence ratio of homoleptic nanocubes (ordered state) is revised (70.7 ± 0.7% yield) according to the six cycles of the transitions between the ordered and disordered states by changing temperature in Figure 3b.

Comment 4:
From the presented data it is also not possible to say how the authors determined the moment when they consider that equilibrium was reached – by the end of visible changes? (which is quite imprecise) or by some numerical method (what was the threshold)? The time of reaching the equilibrium is discussed in many places and the influence of many factors on this value is discussed. Therefore, it is crucial to determine these values in a repeatable way.

Response to the reviewer
We obtained the ^{1}H NMR spectra of the samples that reached equilibration (perfect scrambling between the nanocubes) by annealing the samples in a sealed tube at 130 °C twice. After no change in the ^{1}H NMR spectra was observed, these ^{1}H NMR spectra were used as standard spectra to compare with the ^{1}H NMR spectra measured during the monitor of the scrambling. All the NMR spectra of the equilibrated samples that have been kept at room temperature for about 10 months are the same as was measured after the annealing. As the reviewer pointed out, though we cannot perfectly exclude the possibility that these samples are not in equilibrium, these ^{1}H NMR spectra should be very near to those observed after equilibration.

Comment 5:

Equilibration processes are extremely sensitive to concentration, temperature and additives. The authors did not specify how the temperature was controlled between NMR measurements. Together with imprecise determination of the equilibration timing, these two factors makes the quantitative conclusions very vague.

Response to the reviewer
As the reviewer pointed out, the rate of the scrambling is affected by the temperature. In this study, the temperature of the samples during the scrambling at 25 °C was controlled by thermostat that can keep the target temperature within ± 1°C.

Comment 6:
In the discussion on the influence of added guests on the equilibration process the authors completely ignored the solubility of the guests. The reader can guess that guest TBM is completely insoluble in water, so the equilibration proceeds at the liquid/solid interface, guest PCCP can be slightly soluble in water (although nothing is said about it), while hexane is immiscible in water so equilibration proceeds at the liquid/liquid interface. Therefore, there are three completely different situations with completely different rate limiting factors (phase transfer or even grain size and stirring speed). The authors take these factors into account experimentally and also consider them in the discussion.

Response to the reviewer
As the reviewer pointed out, the encapsulation of the guest molecules (TBM, PCCP, and hexane (Hex)) took place in different situations. Thus the rates of encapsulation of the guest molecules are different. As expected, TBM with extremely low solubility in water was encapsulated most slowly, but PCCP and hexane were encapsulated quickly because small amount of hexane (but this amount is much larger than is required for the encapsulation in the nanocube; the ^{1}H NMR signals of free hexane were observed) is soluble in water, while PCCP is highly soluble in water. However, what is important in this paper is that we mainly discuss the exchanges of GSAs and of guest molecules between the nanocubes after the encapsulation of the guest molecules. Once the guest molecules are encapsulated in the nanocubes, these molecules are isolated in the nanocubes as a small cluster composed of two or three molecules, so these clusters are no longer solid or liquid. It is better to consider the slow exchange of the hydrophobic guest molecules (TBM and hexane) between the nanocubes at the molecular level such as the hydrophobicity of TBM and van der Waals interactions between the guest molecules and the hydrophobic inner surface of the nanocubes.

As to the scrambling between $\mathbf{1}_6$ and TBM$_2$@$\mathbf{2}_6$ in the presence of insoluble TBM, it is true that the encapsulation of TBM may take place between the liquid/solid interface. Another possibility is that a very small amount of TBM molecules solubilized in D_2O (non-detectable level confirmed by ^{1}H NMR) is encapsulated in the nanocubes. Whether which is true or not, what is discussed in this paper is the difference in the rate of scrambling between the two different mixture (a mixture of $\mathbf{1}_6$ and TBM$_2$@$\mathbf{2}_6$ in the presence of insoluble TBM and a mixture of TBM$_2$@$\mathbf{1}_6$ and $\mathbf{2}_6$ in the presence of insoluble TBM). In both cases, the behavior of TBM in these solutions should be almost the same, so whether solid TBM molecules are encapsulated through the liquid/solid interface or solubilized TBM molecules are encapsulated does not affect the rate of scrambling.

Comment 7:
Considering all these reservation the conclusions should be much more balanced.

Response to the reviewer

As we respond to the comments 1–6 described above, the conclusions in this paper are fully based on the experimental results.

Comment 8:
The paper also needs language corrections for improvement of the clarity and general correctness.

Response to the reviewer
The revised manuscript was finally edited by a native English researcher.

To reviewer 3

We are grateful for your careful review and valuable comments. Our response is as follows. Thank you again for your time in advance.

Reviewer 3's comments and our response

Reviewer's Comment

This is an interesting paper in which the scrambling of two self-assembled cubic structures can be controlled through a variety of means--temperature, or neutral and anionic guests. The work is thoroughly done, but I do not think it has the novelty to warrant publication in *Nature Communications*. It is the fourth paper in the series by the authors, detailing further investigations of the systems that were already published and shown to encapsulate guests. In addition, similar guest-modulated transformations have been precedented by e.g. Nitschke, so the phenomenon is not entirely new either. This work is solid, but should be published in a lower-profile journal.

Response to the reviewer

As the reviewer pointed out, we recently published several papers concerning the nanocubes. However, though the thermodynamic property of the nanocubes was discussed in our previous papers, kinetic nature of the nanocubes (kinetic stability and the scrambling property) has not been reported yet. In addition, the main topic in this paper, a cycle of transition between ordered and disordered states by thermal energy, is the first report, though the interconversions by photo- and chemical energies have been reported so far. Thus, we believe that this finding has high enough novelty.

Reviewers' comments:

Reviewer #2 (Remarks to the Author):

Despite the reviewer's requests for more precise data, the authors made only corrections in the text and provided comments. Some of their comments are quite convincing and together with text corrections can be accepted. However, because the authors have not provided new data, I still cannot accept some statements, because I think that they are either very vague or not supported by quantitative data. This stetements should be corrected/explained.

1. Page 3 line 95 "This result suggests that after reaching a statistical mixture of compositional isomers (determined by mass), further scrambling of GSAs in regard to positional isomers took place to reach perfect equilibration (determined by 1H NMR)." The concentrations are slightly but considerably different at MS and NMR experiments and the scrambling process is concentration-dependent d (as the authors proved in previous experiments) Additionally, as it was stated in the original review, the time of equilibration is very imprecisely determined (none of new data have been provided), therefore this statement is not justified.

2. Line 130 „when insoluble TBM existed in an aqueous solution" – what does it mean? As an insoluble solid phase?, what about stirring?

3. Line "insoluble free TBM molecules" – if the molecules are insoluble, they cannot be free in the solution.
4. Line 157 "less stability" – lower stability?

5. To be fully consistent the authors should also provide data for process PCCP2@(2)6 + (1)6 and discuss them.

Response to Reviewers for NCOMMS-18-32696-T

To reviewer 2

We are grateful for your careful review and valuable comments again. Our response is as follows. Thank you again for your time in advance.

Reviewer 2's comments and our response

Comment:

Despite the reviewer's requests for more precise data, the authors made only corrections in the text and provided comments. Some of their comments are quite convincing and together with text corrections can be accepted. However, because the authors have not provided new data, I still cannot accept some statements, because I think that they are either very vague or not supported by quantitative data. This stetements should be corrected/explained.

1. Page 3 line 95 "This result suggests that after reaching a statistical mixture of compositional isomers (determined by mass), further scrambling of GSAs in regard to positional isomers took place to reach perfect equilibration (determined by ^{1}H NMR)." $_1$*The concentrations are slightly but considerably different at MS and NMR experiments and the scrambling process is concentration-dependent (as the authors proved in previous experiments).* Additionally, as it was stated in the original review, $_2$*the time of equilibration is very imprecisely determined (none of new data have been provided), therefore this statement is not justified.*

2. $_3$*Line 130 "when insoluble TBM existed in an aqueous solution" – what does it mean? As an insoluble solid phase?, what about stirring?*

3. $_4$*Line "insoluble free TBM molecules" – if the molecules are insoluble, they cannot be free in the solution.*

4. $_5$*Line 157 "less stability" – lower stability?*

5. $_6$*To be fully consistent the authors should also provide data for process $PCCP_2@\mathbf{2}_6$ + $\mathbf{1}_6$ and discuss them.*

Comment 1:

$_1$The concentrations are slightly but considerably different at MS and NMR experiments and the scrambling process is concentration-dependent (as the authors proved in previous experiments).

Response to the reviewer

^{1}H NMR monitoring of the scrambling between $\mathbf{1}_6$ and $\mathbf{2}_6$ was carried out in the same concentration (0.4 mM) under which the mass measurements were carried out. New data are shown in Figure 3 and Supplementary Figure 3. The scrambling was completed in 2 days in both cases, so the description of this experiment is slightly edited in the revised manuscript.

Comment 2:

$_2$*the time of equilibration is very imprecisely determined (none of new data have been provided), therefore this statement is not justified.*

Response to the reviewer

In order to prove whether a certain state is equilibrated or not, we have no idea except for confirming no change of the spectrum for a long time. In our case, the ^{1}H NMR spectrum of a mixture of $\mathbf{1}_6$ and $\mathbf{2}_6$ standing at 25 °C did not show any change after 2 days. This spectrum is the

same as was measured after standing for 1 and 6 months. It was also confirmed that this spectrum is the same as the ^{1}H NMR spectrum measured after heating in a sealed tube at 130 °C for 2 h. The ^{1}H NMR spectra and the difference NMR spectra are shown below.

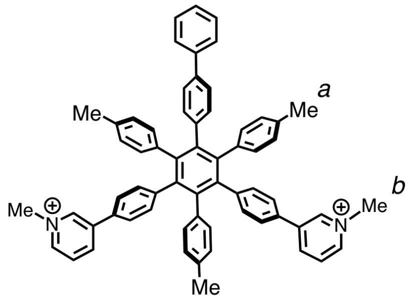

1^{2+}

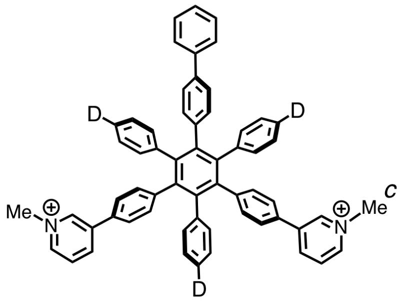

2^{2+}

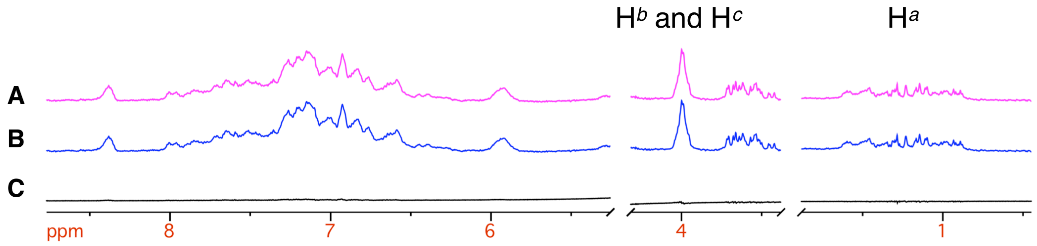

A. ^{1}H NMR spectrum of a mixture of $\mathbf{1}_6$ and $\mathbf{2}_6$ after standing at 25 °C for 1 month. **B.** ^{1}H NMR spectrum of a mixture of $\mathbf{1}_6$ and $\mathbf{2}_6$ after standing at 25 °C for 2 days, when the spectral change converged. **C.** difference ^{1}H NMR spectrum prepared by subtracting **B** from **A**. ^{1}H NMR spectra were measured at 298 K in D_2O.

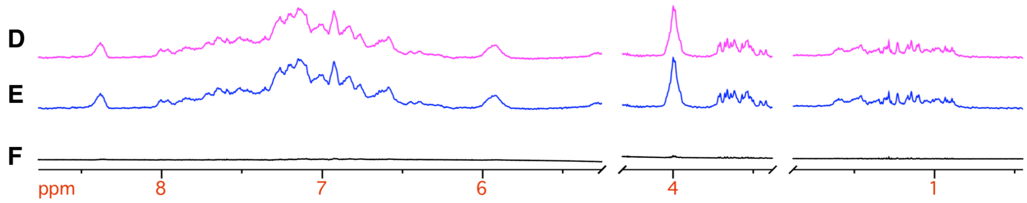

D. ^{1}H NMR spectrum of a mixture of $\mathbf{1}_6$ and $\mathbf{2}_6$ after standing at 25 °C for 6 month. **E.** ^{1}H NMR spectrum of a mixture of $\mathbf{1}_6$ and $\mathbf{2}_6$ after standing at 25 °C for 2 days, when the spectral change converged. **F.** difference ^{1}H NMR spectrum prepared by subtracting **E** from **D**. ^{1}H NMR spectra were measured at 298 K in D_2O.

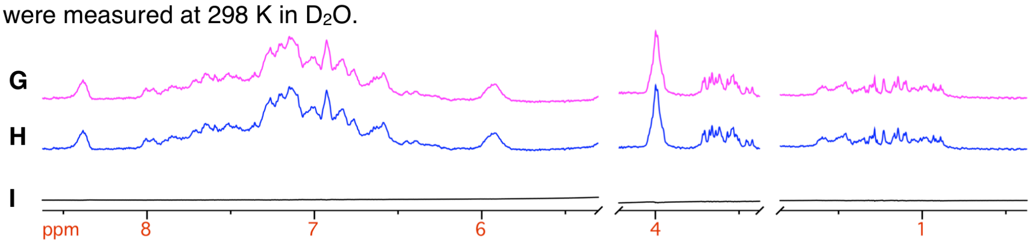

G. ^{1}H NMR spectrum of a mixture of $\mathbf{1}_6$ and $\mathbf{2}_6$ after heating at 130 °C for 2 h. **H.** ^{1}H NMR spectrum of a mixture of $\mathbf{1}_6$ and $\mathbf{2}_6$ after standing at 25 °C for 2 days, when the spectral change converged. **I.** difference ^{1}H NMR spectrum prepared by subtracting **H** from **G**. ^{1}H NMR spectra were measured at 298 K in D_2O.

In addition, the decrease in the $\mathbf{1}_6$ nanocube, whose consumption is slower than that of $\mathbf{2}_6$, is analyzed. As the figure shown in below indicates, most of the $\mathbf{1}_6$ was consumed within 2 days. Considering that a small amount of $\mathbf{1}_6$ should remain after the equilibration, under which an almost statistical mixture of the nanocubes were produced, it is confirmed that the scrambling reached convergence in 2 days. Since many heteroleptic species are produced by the scrambling, it is quite difficult to establish an exact kinetic model for the scrambling. Thus, the decrease in the concentration of $\mathbf{1}_6$ was simply fitted by a first-order equation, which is indicated as a black curve.

The reason why the plot at 0 h is out of the fitting curve is because in the beginning of the scrambling less stable $\mathbf{2}_6$ nanocube mainly participated in the scrambling.

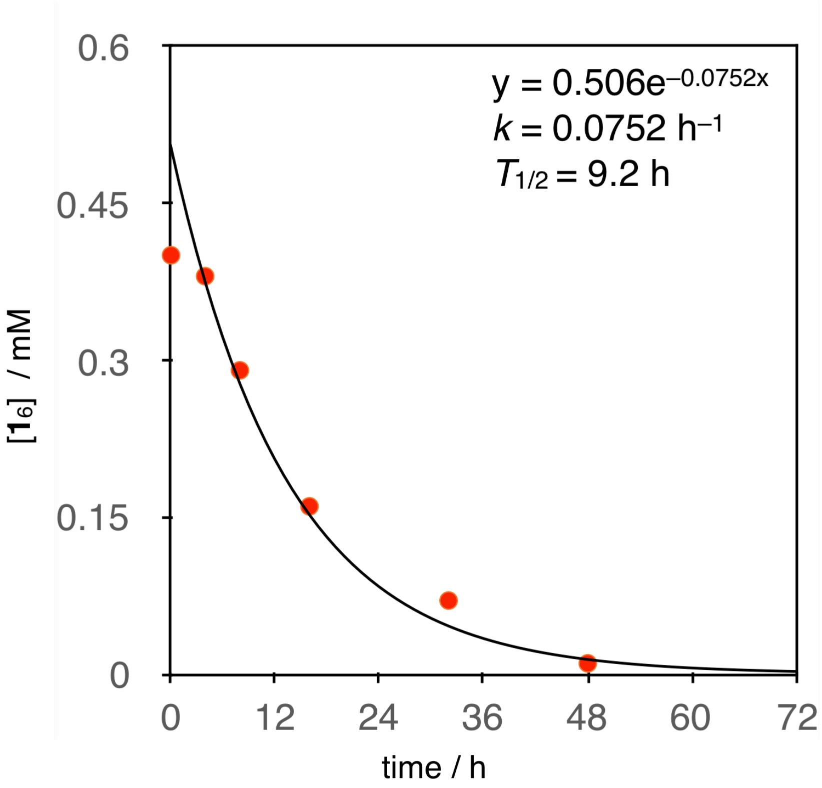

Figure. Red solid circles indicate the change in the concentration of $\mathbf{1}_6$ during the scrambling between $\mathbf{1}_6$ and $\mathbf{2}_6$ in D_2O at 298 K. The concentration was determined by the integration of the ^{1}H NMR signal of $\mathbf{1}_6$ ([**1**] = [**2**] = 0.4 mM). A black line indicates the fitting curve for the data.

Comment 3:

3*Line 130 "when insoluble TBM existed in an aqueous solution" – what does it mean? As an insoluble solid phase?, what about stirring?*

Response to the reviewer

What this sentence means that because TBM is not soluble in water, particles of TBM exists in the solution. The ^{1}H NMR spectrum of solid TBM in D_2O shows no peaks except for the solvent peak. The encapsulation of TBM in the nanocube would take place on the interface of the particles (TBM) and D_2O phase when dissociated GSAs diffuse to the surface of TBM solid (though we do not have any experimental evidence to prove this idea). The scrambling experiment was carried out without stirring.

Comment 4:

4*Line "insoluble free TBM molecules" – if the molecules are insoluble, they cannot be free in the solution.*

Response to the reviewer

"free" in this sentence "*Insoluble free TBM molecules were gradually encapsulated in* $\mathbf{1}_6$*, during which the structure of the* $\mathbf{1}_6$ *nanocube would partially be broken or free GSAs would be*

dissociated." means that TBM is not encapsulated in the nanocube like "free guest molecule", as is often used. To avoid confusion, "free" is removed in the revised manuscript.

Comment 5:

5 *Line 157 "less stability" – lower stability?*

Response to the reviewer

According to the reviewer's kind suggestion, the sentence is revised.

Comment 6:

6 *To be fully consistent the authors should also provide data for process* $PCCP_2@\mathbf{2}_6 + \mathbf{1}_6$ *and discuss them.*

Response to the reviewer

The experimental result for the scrambling between $PCCP_2@\mathbf{2}_6$ and $\mathbf{1}_6$, which is perfectly the same as the scrambling experiment between $\mathbf{1}_6$ and $\mathbf{2}_6$ in the presence of PCCP, is added in the revised manuscript (Supplementary Fig. 14).

REVIEWERS' COMMENTS:

Reviewer #2 (Remarks to the Author):

After additional experiments and satisfactory explanations by the authors I am glad to recommend publication of the paper in this journal.
